# Progerin Hinders Autophagy Flux at Its Final Stages in Hutchinson‐Gilford Progeria Syndrome Cells, Preventing Its Own Autophagic Degradation

**DOI:** 10.1111/acel.70649

**Published:** 2026-07-29

**Authors:** Ian García‐Aguirre, Jesús Alejandro Reyes‐Martínez, Juan Unzueta, Francisco Guevara‐Namorado, Solangy Lizcano‐Meneses, Susana Gonzalo, Angel Baldan, Claudia Rangel, Gerardo J. Alanis‐Funes, Francisco Garcia‐Sierra, Kevin Ruiz‐Fajardo, Simon Gormes‐Pinchanski, Susana Castro‐Obregón, Aranza Meza‐Dorantes, Rocio Alejandra Chavez‐Santoscoy, Isabel Arrieta‐Cruz, Paola Tristán‐Aburto, Jonathan J. Magaña, Bulmaro Cisneros

**Affiliations:** ^1^ Departamento de Bioingeniería Escuela de Ingeniería y Ciencias, Tecnologico de Monterrey Ciudad de México Mexico; ^2^ Departamento de Genética y Biología Molecular Centro de Investigación y de Estudios Avanzados Ciudad de México México; ^3^ Department of Cell Biology Center for Research and Advanced Studies of the National Polytechnic Institute Mexico City Mexico; ^4^ Edward A. Doisy Department of Biochemistry and Molecular Biology Saint Louis University School of Medicine St. Louis Missouri USA; ^5^ Escuela de Ingeniería y Ciencias, Tecnológico de Monterrey Monterrey Mexico; ^6^ Computational and Integrative Biology National Institute of Genomic Medicine Mexico City Mexico; ^7^ School of Engineering and Sciences Tecnologico de Monterrey, Campus Querétaro Querétaro Mexico; ^8^ Instituto de Fisiología Celular, UNAM Ciudad Universitaria Ciudad de México México; ^9^ División de Investigación, Departamento de Investigación Básica Instituto Nacional de Geriatría, Secretaría de Salud Ciudad de México Mexico; ^10^ Laboratorio de Medicina Genómica, Departamento de Genética (CENIAQ) Instituto Nacional de Rehabilitación‐Luis Guillermo Ibarra Ibarra (INR‐LGII) Ciudad de México Mexico

**Keywords:** aging, autophagy, lysosomes, progeria

## Abstract

In Hutchinson‐Gilford progeria syndrome (HGPS), dysfunctional autophagy results in the accumulation of progerin, a lamin A mutant variant that alters a plethora of processes, inducing senescence and driving premature aging. Therefore, the elimination of progerin through autophagy restoration emerges as a therapeutic intervention against HGPS. However, a comprehensive study of autophagy flux in HGPS remains to be addressed. In this study, the dynamics of autophagy in HGPS fibroblasts were analyzed utilizing different HGPS cell models and experimental approaches. The autophagy‐associated transcriptomic profile was determined, and the autophagy‐lysosome axis was comprehensively analyzed. We demonstrated that progerin induces the formation of autophagosomes but impairs their maturation and subsequent fusion with lysosomes. This alteration is attributed in part to the progerin‐mediated decreased expression of STX17, a marker of mature autophagosomes, and LAMP1, a membrane lysosomal protein, as well as the presence of defective lysosomes. In line with this, the rescue of STX17 and LAMP1 expression improved autophagy flux. Interestingly, treatment of HGPS fibroblasts with Selinexor, an autophagy activator, elicited nuclear accumulation of TFEB and enhanced lysosomal biogenesis and function, thereby activating autophagy. Selinexor treatment improved both autophagosome maturation and autophagosome‐lysosome fusion, which ultimately led to effective autophagic degradation of progerin. In summary, progerin impedes proper autophagy flux, thus preventing its own autophagic degradation, which underscores the relevance of targeting the autophagy‐lysosome pathway to counteract the toxic accumulation of progerin.

## Graphical abstract

We demonstrated that progerin induces the formation of autophagosomes but impairs their maturation and subsequent fusion with lysosomes. Progerin impedes proper autophagy flux, thus preventing its own autophagic degradation. Selinexor treatment improved both autophagosome maturation and autophagosome‐lysosome fusion, which ultimately led to effective autophagic degradation of progerin.

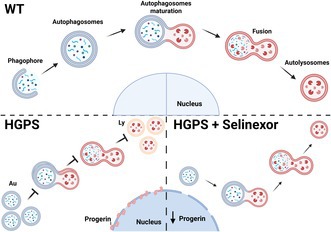

The study of Hutchinson‐Gilford progeria syndrome (HGPS) has led to a significant advance in the understanding of aging. This rare condition is caused by a point mutation that uncovers a cryptic splicing site in exon 11 of the lamin A gene. This results in the removal of 50 amino acids near the C‐terminus of prelamin A, thereby yielding a permanently farnesylated and carboxymethylated protein termed progerin. Progerin has been demonstrated to disrupt nuclear envelope (NE) organization, consequently impairing a range of aging‐related mechanisms (De Sandre‐Giovannoli et al. 2003; Desai et al. 2025; Eriksson et al. 2003). Specifically, the failure of autophagy, a cytoprotective process that allows the elimination of damaged organelles and long‐lived/misfolded proteins to recycle essential components (Aman et al. 2021; Kaushik et al. 2021), is believed to result in the accumulation of progerin, which triggers in turn cellular senescence (Borroni et al. 2018; Son et al. 2024). Thus, a viable therapeutic approach involves the activation of autophagy to facilitate the clearance of progerin (Aveleira et al. 2020; Ferreira‐Marques et al. 2023; Gabriel et al. 2015; Son et al. 2024). However, a systematic analysis of the autophagy flux to determine at which step failure occurs is lacking.

In this study, we provide a detailed multi‐level analysis of autophagy flux in HGPS fibroblasts using various cellular models and experimental strategies. The analysis encompassed the formation and maturation of autophagosomes, lysosome morphology and function, the autophagosome‐lysosome fusion, the lysosomal turnover of the autolysosome content, and analysis of autophagy flux with a retroviral autophagy probe. Firstly, the initial phase of autophagy was analyzed in primary fibroblasts derived from HGPS patients by evaluating the number of autophagosomes and their content. HGPS‐1/‐2 cells were treated with chloroquine (CQ) for 48 h to block the autophagy flux and enable the quantification of accumulated autophagosomes using microtubule‐associated protein 1A/1B light chain 3 (LC3), a *bona fide* marker of autophagosome formation (Itakura and Mizushima 2010). Both increased LC3‐labeled fluorescent foci and augmented conversion of LC3‐I to LC3‐II (LC3‐phosphatidylethanolamine conjugation) were found in HGPS‐1/2 fibroblasts treated with CQ by confocal microscopy and immunoblotting assays respectively (Figure 1A,B). However, CQ treatment for 12 h revealed a delay in the accumulation of LC3‐immunostained autophagosomes in HGPS‐1 fibroblasts compared to WT cells (Figure S1). On the other hand, autophagic vacuoles quantified by Cyto‐ID labeling (Figure 1C) as well as the immunofluorescent signal and protein levels of p62/SQSTM1 (Figure S2), a selective receptor and substrate of autophagy, increased significantly in CQ‐treated HGPS‐1‐cells, compared to CQ‐treated WT cells. Overall, these results indicate that autophagy is basally activated in HGPS fibroblasts, but with evidence of impaired lysosomal turnover, suggesting that flux is not fully efficient. Nonetheless, the conversion of LC3‐I to LC3‐II was significantly reduced in HGPS‐1 fibroblasts cultured under starvation conditions and treated with the inhibitors of lysosomal function E64D and pepstatin A, compared with WT cells (Figure 1D). This implies that the lysosomal turnover of LC3‐II is impaired due to an impediment in the final stages of autophagy flux, likely due to impaired clearance. Therefore, a direct assessment of the impact of progerin on autophagy flux was undertaken. BJ fibroblasts stably expressing progerin or the empty vector were retrovirally transduced to express the autophagy flux probe GFP‐LC3‐RFP‐LC3ΔG, which allows for a reliable measurement of autophagy activity (Kaizuka et al. 2016). This chimeric protein undergoes cleavage by intracellular autophagy related gene 4 (ATG4) proteases yielding GFP‐LC3 and RFP‐LC3DG. Under starvation conditions, GFP‐LC3 is then degraded by autophagy, while RFP‐LC3ΔG remains intact and serves as an internal control. Induction of autophagy was observed in BJ cells after 12 h of starvation, as indicated by a decrease in both the GFP‐LC3 fluorescent signal and the LC3‐II electrophoretic band (Figure 1E,F). Conversely, the expression of progerin in these cells resulted in enhanced GFP‐LC3 fluorescence and the accumulation of the LC3‐II band (Figure 1E,F), indicating the blockage of autophagy. Treatment of fibroblasts for 6 days with an autophagy inducer, Selinexor (Silvestrini et al. 2018), was shown to restore autophagy flux in progerin‐expressing fibroblasts as demonstrated by the decrease of both GFP‐LC3 fluorescence and the LC3‐II band at 12 h of starvation. This resulted in progerin clearance (Figure 1E,F; compare 0 h versus 12 h of starvation with or without Selinexor). The quantification of confocal microscopy images and the cytometry flux analysis of autophagy fluorescent probes confirmed these observations (Figure S3). These findings collectively indicate that progerin expression indeed hinders autophagy flux and that Selinexor counteracts this effect, promoting autophagy‐mediated progerin degradation.

**FIGURE 1:**
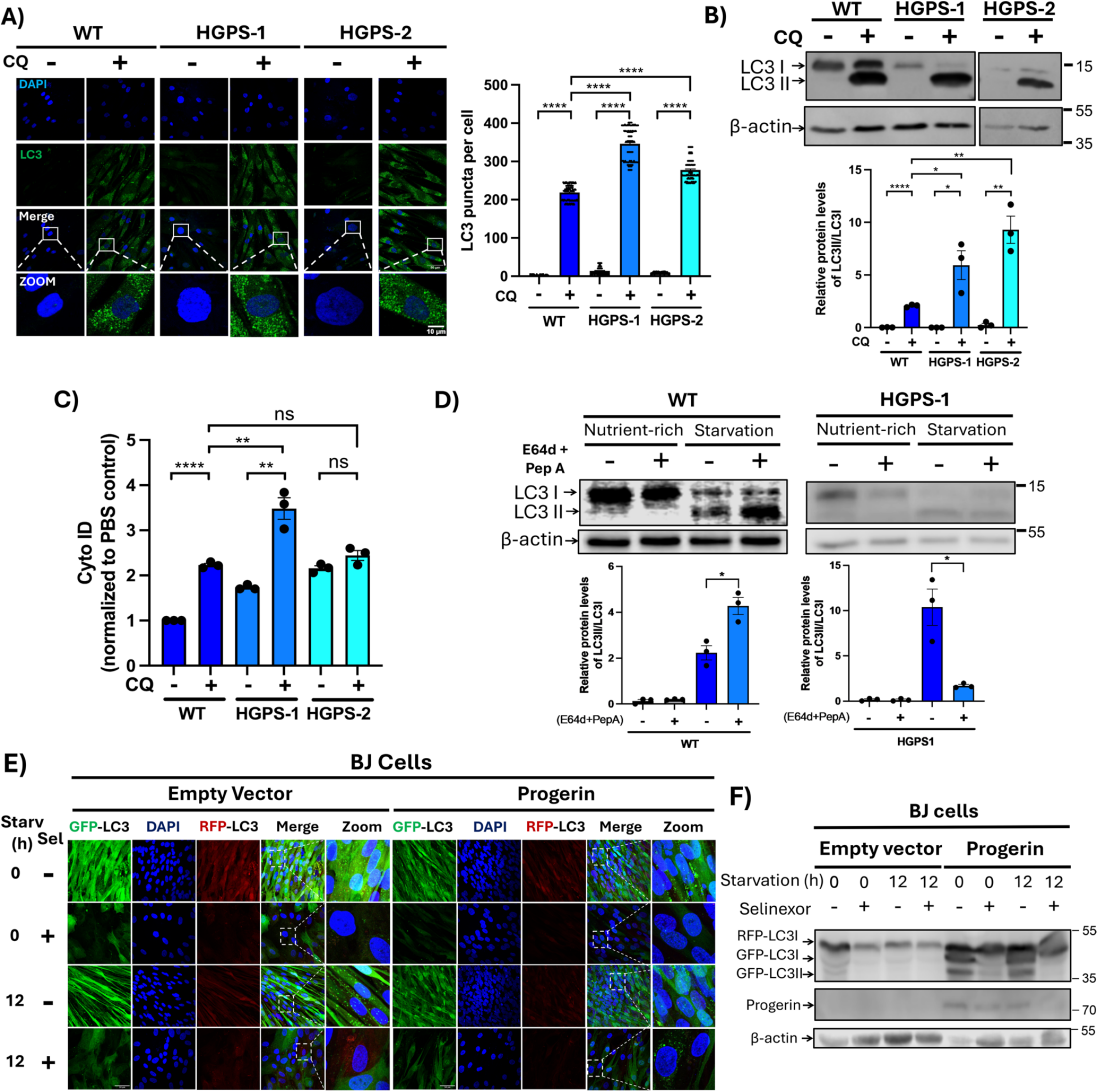
Autophagy flux is impaired in HGPS fibroblasts, and its restoration via Selinexor treatment promotes progerin clearance. (A) Autophagosomes accumulate in HGPS fibroblasts. The indicated cell cultures were treated with CQ prior to immunofluorescence staining for LC3. *Right*. The number of LC3 puncta per cell was determined. (B) The conversion of LC3‐I to LC3‐II is increased in HGPS fibroblasts. The indicated cell cultures were treated with CQ and analyzed by immunoblotting for LC3. *Bottom*. The LC3‐II/LC3‐I ratio was measured. (C) Autophagic vacuoles were estimated in HGPS fibroblasts. The indicated cell cultures were treated with CQ prior to being stained with the Cyto‐ID autophagy detection kit and analyzed by flow cytometry. (D) Lysosomal turnover of LC3‐II is diminished in HGPS‐1 cells. The indicated cell cultures were incubated with KRB medium (starvation) and treated with the lysosomal protease inhibitors E64 and pepstatin A, prior to Western blotting analysis for LC3. *Bottom*. The LC3II/LC3I lysosomal ratio was estimated. (E) BJ cells harboring an empty vector or a vector expressing progerin were transduced with an autophagy probe (GFP‐LC3‐RFP‐LC3ΔG) to measure autophagy flux. The cells grown in KRB medium (starvation) were treated with Selinexor for 6 days when indicated, and further analyzed by confocal microscopy. (F) BJ cell lysates previously treated as per E were analyzed by Western blotting for progerin and the indicated LC3‐fusion proteins. See the Supplementary Figure Legends for details.

To investigate how progerin expression alters autophagy flux, a transcriptome profiling of WT and HGPS fibroblasts grown under basal conditions (nutrient‐rich medium) was carried out using high‐throughput RNA sequencing (RNA‐seq). The principal component analyses (PCA) successfully separate WT from HGPS‐1 fibroblasts (Figure S4 and Methods S1). By comparing the transcriptome profile of WT and HGPS‐1 fibroblasts, we identified 7891 differentially expressed genes (DEGs) in HGPS fibroblasts: 3983 were upregulated and 39,089 were downregulated (Figures S4B,C and S5A). Gene set enrichment analysis revealed the top 10 cellular processes enriched in HGPS fibroblasts when contrasted with WT cells, being lysosomes and autophagy the second and fourth most enriched processes (Figure 2A). Specifically, a number of differentially expressed genes (DEGs) related to autophagy (Figure S5B), targeted by TFEB, including VPS18, CTSA, and CTSD (Figure 2B), or associated with lysosomal function (Figure 2C and Figure S5C), were found to be significantly decreased in HGPS fibroblasts. Collectively, these results suggest an altered autophagy‐lysosome axis function in HGPS fibroblasts. Because transcription factor B (TFEB) is a master regulator of autophagy and lysosomal biogenesis (Abokyi et al. 2023), its state was evaluated in HGPS fibroblasts. Under physiological conditions, the phosphorylated, inactive form of TFEB is predominantly located in the cytoplasm. However, under conditions of stress, TFEB is activated by calcineurin‐mediated dephosphorylation, resulting in its subsequent translocation to the nucleus and the upregulation of its target genes (Li et al. 2019). We found that levels of phospho‐TFEB (p‐TFEB) decreased markedly in both WT and HGPS fibroblasts upon autophagy induction with KRB‐mediated nutrient starvation, indicating that activation of TFEB occurs with similar efficacy between the two cell types (Figure 2D). Consistently, confocal microscopy analysis revealed a clear nuclear accumulation of TFEB in the nucleus of both WT and HGPS‐1 fibroblasts upon KRB‐mediated nutrient starvation (Figure 2E). Therefore, the TFEB‐mediated transcriptional control of autophagy appears to be unaltered in HGPS fibroblasts. However, downregulation of TFEB target genes in HGPS‐1 fibroblasts (see above) is inconsistent. It can be argued that besides phosphorylation, the activity of TFEB is modulated by competitive DNA binding to CLEAR (Coordinated Lysosomal Expression and Regulation) motifs with repressors (USF2) and by chromatin accessibility to CLEAR (Chen et al. 2024).

**FIGURE 2:**
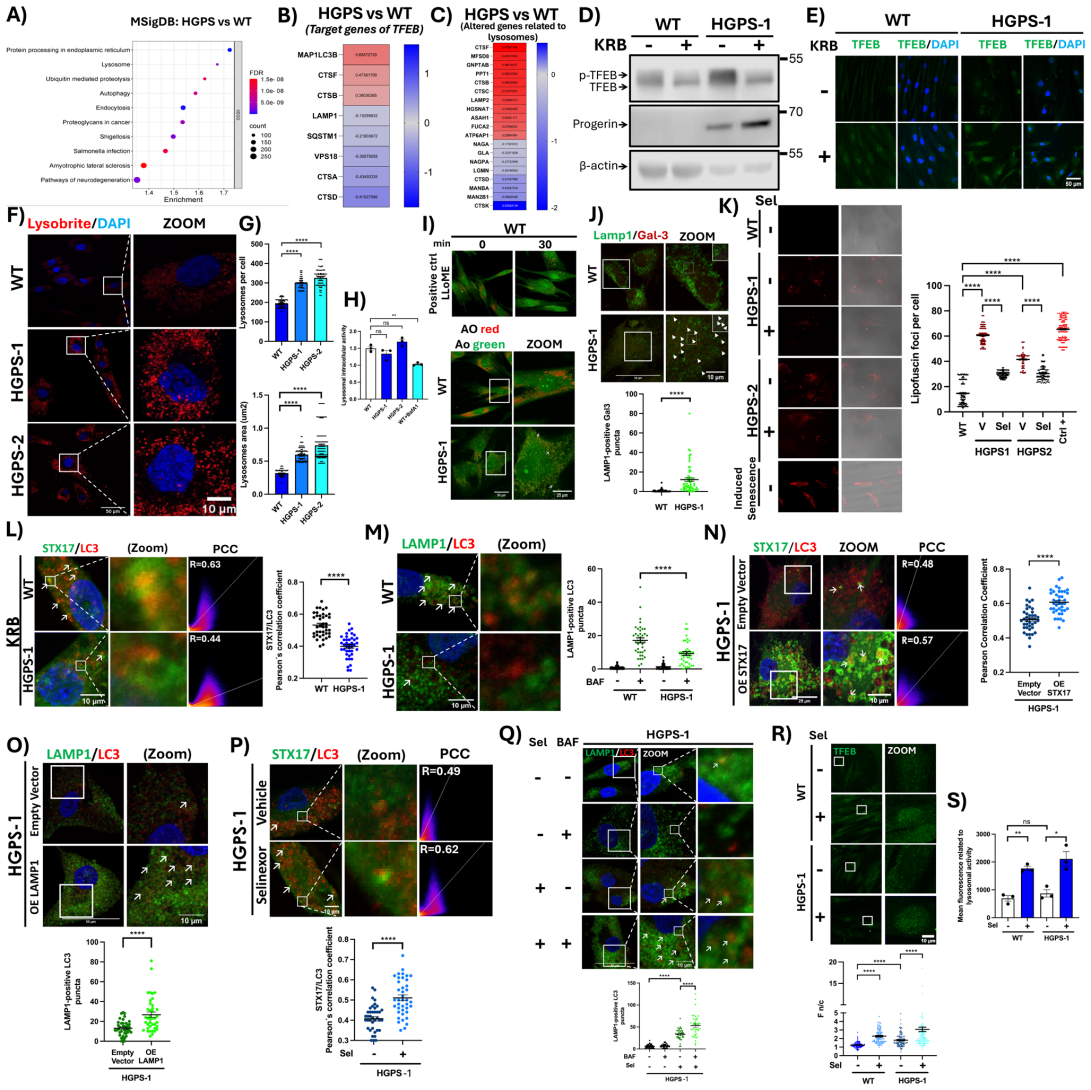
Impediment of autophagy in late stages in HGPS fibroblasts and its release by Selinexor treatment. (A) The top ten Gene Ontology biological processes enriched in HGPS fibroblas. (B and C) The DEGs in HGPS‐1 cells that are targeted by TFEB (B) or associated with lysosomal function (C) are displayed. (D) The phosphorylation state of TFEB was analyzed by Western blotting in HGPS fibroblasts grown in KRB medium, (E) KRB‐mediated starvation induces the nuclear accumulation of TFEB in HGPS‐1 cells, as shown by confocal microscopy analysis. (F) Lysosome analysis in HGPS fibroblasts using LysoBrite staining and confocal microscopy. (G) The number of lysosomes per cell and per area (Bottom) were quantified. (H) The lysosomal intracellular activity was assessed in HGPS fibroblasts by flow cytometry. (I and J) Lysosomal membrane permeabilization analyzed by acridine orange (AO) (I) and Galectin‐3 (J) assays, *Down*. The number of LAMP1‐Gal3 positive puncta was quantified. Treatment with LLoMe (LMP inductor) for 30 min was used as positive control. (K) The analysis of lipofuscin aggregates was conducted in HGPS fibroblasts treated with Selinexor (Sel). *Right*. The number of lipofuscin foci per cell was estimated. (L) To evaluate the maturation of autophagosomes, the colocalization of LC3 and STX17 was analyzed by confocal microscopy. *Right*. The Pearson correlation coefficient (PCC) for the STX17‐LC3 colocalization was obtained. (M) To assess the fusion of autophagosomes and lysosomes, the colocalization of LC3 and LAMP1 was examined by confocal microscopy. *Right*. The number of LAMP1‐LC3 positive puncta was quantified. (N) The effect of rescuing STX17 expression on autophagosome maturation was analyzed in CQ‐treated HGPS fibroblasts grown under starvation. The Pearson correlation coefficient (PCC) for the STX17‐LC3 colocalization was obtained. (O) The impact of rescuing LAMP1 expression by viral transduction on autophasome‐lysosome fusion was evaluated in Bafilomycin‐treated HGPS fibroblasts. The quantity of LAMP1‐LC3 positive puncta was determined. (P) The effect of Selinexor treatment on the LC3 and STX17 colocalization in HGPS fibroblasts. *Bottom*. The Pearson correlation coefficient (PCC) for the STX17‐LC3 colocalization was obtained. (Q) The impact of Selinexor treatment on the colocalizing of LC3 and LAMP1 is shown. *Bottom*. The quantity of LAMP1‐positive LC3 puncta was determined. (R) Selinexor treatment induces the nuclear accumulation of TFEB in HGPS‐1 fibroblasts, as shown by confocal microscopy analysis. *Bottom* The nuclear to cytoplasmic ratio of fluorescence (Fn/c) was obtained. (S) Selinexor treatment induces the lysosomal activity in WT and HGPS‐1 fibroblasts, as shown by flow cytometry. See the Supplementary Figure Legends for details.

Since lysosomes are critical for the completion of the autophagy flux (Yang and Zhang 2023; Yim and Mizushima 2020), an evaluation of the lysosomal morphology and function was then conducted in HGPS fibroblasts. A significant increase in the abundance and size of lysosomes was observed in HGPS‐1/‐2 cells compared to WT cells, as demonstrated by LysoBrite staining and subsequent quantification (Figure 2F,G). It is worth noting that expanded lysosomes are indicative of lysosomal dysfunction (Tan and Finkel 2023; Yim and Mizushima 2020). While the level of intracellular lysosomal activity was comparable between WT and HGPS fibroblasts (Figure 2H). Lysosomal membrane permeabilization (LMP), a critical parameter of lysosomal function and integrity. Thus, LMP was analyzed using Acridine Orange (AO) and Galectin‐3 (Gal3) assays. LMP was present in HGPS‐1 fibroblasts as demonstrated by the decrease of the red fluorescence of AO‐stained lysosomes (indicative of functional lysosomes) and the increase in the binding of Galectin to the lysosomal membrane (indicative of damaged lysosomal membrane), compared with WT fibroblasts (Figure 2I,J and Figure S6A,B). In line with this notion, HGPS lysosomes exhibited increased autofluorescence of lipofuscin (oxidized lipid and protein complexes) (Figure 2K). Overall, these findings indicate that HGPS lysosomes are permeable/impaired and overloaded with waste material and damaged cellular components. In line with this, a recent study revealed the presence of defective lysosomes in fibroblasts from HGPS patients (Wang et al. 2025). As the presence of defective autophagosome‐lysosome axis can ultimately affect the completion of autophagy (Tan and Finkel 2023; Yim and Mizushima 2020), an evaluation of the late stages of autophagy, namely autophagosome maturation and autophagosome‐lysosome fusion, was undertaken. The recruitment of the autophagosomal Soluble N‐ethylmaleimide‐sensitive factor activating protein receptor (SNARE) protein STX17 to sealed autophagosomes is critical for their maturation (Itakura et al. 2012; Itakura and Mizushima 2013); thus, its colocalization with LC3 was evaluated by confocal microscopy in fibroblasts cultured under starvation condition and treated with CQ. LC3 puncta colocalizing with STX17 significantly decreased in HGPS fibroblasts (Figure 2L), as shown by Pearson correlation value (Right graph, 0.63 vs. 0.44 for WT and HGPS‐1 fibroblasts, respectively). Likewise, colocalization of LC3 with the lysosomal membrane protein LAMP1 was evaluated as an indicator of autophagosome‐lysosome fusion frequency. WT and HGPS‐1 fibroblasts were treated with bafilomycin A1 (BAF) to inhibit vacuolar‐type H^+^‐ATPase (V‐ATPase)‐dependent acidification of lysosomes (Mauvezin and Neufeld 2015) and favor the accumulation of fused autophagosome‐lysosome structures prior to confocal microscopy analysis. Consistent with the hypothesis of impaired autophagy in late stages, the number of LC3 puncta colocalizing with LAMP1 decreased significantly in HGPS fibroblasts, compared to WT cells (Figure 2M). The decline in autophagy maturation and autophagosome‐lysosome fusion is correlated with a reduction in STX17 and LAMP1 proteins respectively. Because a decrease in STX17 and LAMP1 immunostaining was observed in both HGPS‐1/‐2 fibroblasts (Figure S7A,B) and GFP‐progerin expressing HDFs (Figure S8A,B). Concurrently, reduced STX17 and LAMP1 protein levels were evidenced in HGPS‐2 fibroblasts (Figure S7C). Furthermore, the expression of GFP‐progerin in HDFs resulted in mislocalization of STX17 to the nucleus (Figure S8B). The rescue of STX17 and LAMP1 expression using lentiviral transduction resulted in enhanced autophagosome maturation (Figure 2N) and autophagosome‐lysosome fusion (Figure 2O) in HGPS‐1 fibroblasts grown under starvation conditions, respectively. Thus, proper expression of these proteins is critical for optimal autophagy flux. Remarkably, treatment of HGPS‐1 fibroblasts with Selinexor for three or six days led to an enrichment of LC3‐STX17 (Figure 2P and bottom graph, and Figure S9) and LC3‐LAMP1 punctuate colocalization (Figure 2Q and bottom graph, and Figure S10). In addition, Selinexor treatment resulted in higher nuclear accumulation of TFEB (Figure 2R and bottom graph), increased lysosomal activity (Figure 2S) and lesser accumulation of lipofuscin (Figure 2K and right graph). It is worth noting that Selinexor treatment neither induced apoptosis nor necrosis in WT and HGPS‐1 fibroblasts (Figure S11).

Collectively these data suggested the efficacy of Selinexor in unblocking the final steps of autophagy flux in HGPS fibroblasts and consequently promotes the clearance of progerin (Figure 1E,F).

The decline in autophagy in HGPS fibroblasts has been attributed mainly to the overactivation of mammalian target of rapamycin complex 1 (mTORC1) (Cao et al. 2011; Ferreira‐Marques et al. 2023; Ferret et al. 2024; Son et al. 2024). mTORC1 complex is a key regulator of cell growth and proliferation in response to nutrient signaling (Kim and Guan 2015), which concurrently inhibits autophagy initiation through the phosphorylation of diverse components of the ULK complex, including autophagy related gene 13 (ATG13) and Unc‐51‐like autophagy‐activating kinase 1 (ULK1) and autophagy/beclin 1 regulator 1 (AMBRA1) (Ganley et al. 2009; Hosokawa et al. 2009; Kim and Guan 2015). A recent study demonstrated that hyperactivation of mTORC1 in HGPS fibroblasts is triggered by an abnormal accumulation of the acetyl transferase EP300 in the cytoplasm (Ferret et al. 2024; Son et al. 2024), due to the exacerbated activity of exportin CRM1 present in HGPS fibroblasts (García‐Aguirre et al. 2019). It was shown by these authors that an excess of cytoplasmic p300 promotes the acetylation of Raptor, thereby facilitating the binding of mTORC1 to the lysosome, a necessary step for mTORC1 activation (Cao et al. 2011; Son et al. 2024). Furthermore, mTORC1 negatively regulates autophagy by phosphorylating TFEB at Ser 142 and Ser 211, thereby promoting its sequestration in the cytoplasm (Martina et al. 2012; Napolitano et al. 2018). Therefore, the determination of mTORC1 overactivation by analyzing the phosphorylation state of mTORC1 components (mTOR, pS6K1, 4E‐BP1), has been invoked as evidence of autophagy initiation inhibition (Cao et al. 2011; Ferreira‐Marques et al. 2023; Ferret et al. 2024; Son et al. 2024). Conversely, we observed higher TFEB nuclear accumulation and increased LC3‐II levels in HGPS fibroblasts which is consistent with activation of autophagy initiation. Instead, the failure of autophagy flux in HGPS fibroblasts was found to occur mainly at the late stages, namely autophagosome maturation and its fusion with the lysosome. In concordance, a progerin‐mediated autophagy activation with a further blockage of the autophagy flux was found in HDFs that expressed progerin in an inducible manner (Cancado de Faria et al. 2023). In alignment with our hypothesis, treatment of HGPS fibroblasts with Selinexor induced progerin clearance by improving the late stages of autophagy, likely through the promotion of TFEB nuclear accumulation and transcriptional activity, as well as the enhancement of lysosomal biogenesis and activity. It is worth noting that Selinexor is a broad inhibitor of nuclear protein export, thus the accumulation of numerous CRM1 target proteins in the nucleus following treatment would result in complex regulation. For instance, we demonstrated that Selienxor treatment resulted in the nuclear accumulation of p53 and FOXO3 in WT and HGPS‐1 fibroblasts (Figure S12). Further analysis is necessary to determine whether Selinexor treatment rescues the expression of TFEB‐target genes that were found downregulated in HGPS‐1 fibroblasts. We demonstrated that the activation of autophagy induced by Selinexor occurs independently of mTOR signaling. The phosphorylation state of S6, a ribosomal protein that undergoes phosphorylation by S6K1 in nutrient‐rich conditions, exhibited a drastic decline in HGPS‐1 fibroblasts grown in starvation conditions, which is compatible with inactivation of mTOR and consequently activation of autophagy. The levels of pS6 remained undetectable following six days of Selinexor treatment (Figure S13A,B). In the context of autophagy‐mediated progerin clearance being one of the most viable therapies against HGPS, this study provides evidence that progerin impedes autophagy at the late stages to protect itself from autophagic degradation, which would prompt the development of strategies to enhance autophagy flux specifically at the final steps.

## Author Contributions

Conceptualization: I.G.‐A. and B.C. Data curation: B.C., I.G.‐A., J.U., J.A.R.‐M., F.G.‐N., S.L.‐M., C.R., G.J.A.‐F., K.R.‐F. and A.M.‐D. Formal Analysis: B.C., I.G.‐A., J.U., J.A.R.‐M., F.G.‐N., S.L.‐M., C.R. and G.J.A.‐F. Funding Acquisition: B.C., I.G.‐A., S.G., A.B., J.J.M. and I.A.‐C. Investigation: I.G.‐A., B.C., J.U., J.A.R.‐M., F.G.‐N., S.L.‐M., C.R., G.J.A.‐F., K.R.‐F., A.M.‐D., S.G., P.T.‐A. and A.B. Project Administration: B.C., I.G.‐A. and J.J.M. Resources: B.C., I.G.‐A., J.J.M., S.G., A.B., F.G.‐S. and I.A.‐C. Supervision: B.C., I.G.‐A. and S.G. Visualization: B.C., I.G.‐A. and S.C.‐O. Wrting – original draft preparation: B.C. and I.G.‐A. Writing – review and editing: B.C., I.G.‐A., S.G. and S.C.‐O.

## Funding

This research supported by Challenge‐Based Research Funding Program ITESM grant number CI_EIC_HLT_D_208 (to I.G.‐A. and J.J.M.), Ciencia básica y de Frontera. SECIHTI. No. CBF‐2025‐G‐806 (to B.C.) and by ITESM Grant (2021Tec‐BASE Seed Fund for Research Projects) (to J.J.M.).

## Conflicts of Interest

The authors declare no conflicts of interest.

## Supporting information


**Figure S1:** A delay in the formation of autophagosomes in HGPS‐1 fibroblasts. (A and B) WT and HGPS‐1 fibroblasts grown on coverslips were treated with 50 μM CQ for 12 h prior to immunofluorescence staining for LC3 (A) and p62 (B). The fibroblasts were counterstained with DAPI to decorate the nuclei. Representative images of confocal microscopy from three separate experiments are shown. Bottom. The number of LC3 (A) and p62 (B) puncta per cell were quantified (*n* = 30 cells per experimental condition) using NIS elements software (Nikon), with significant differences determined by Mann–Whitney (****p < 0.0001).
**Figure S2:** Accumulation of SQSTM1/p62 protein in WT and HGPS fibroblasts upon CQ‐mediated autophagy inhibition. (A) WT and HGPS‐1/‐2 fibroblasts grown on coverslips were treated with 50 μM CQ or the vehicle alone for 48 h and then double immunostained for LC3 and p62. Nuclei were labeled with DAPI for visualization prior to confocal microscopy analysis. (B) Lysates from WT and HGPS‐1/HGPS‐2 cultures treated with CQ as per A were subjected to western blotting analysis using antibodies specific to p62 and β‐actin (loading control).
**Figure S3:** Quantitative analysis of the autophagy flux probe GFP‐LC3‐RFP‐LC3ΔG in BJ fibroblasts expressing the empty vector or progerin. (A) The GFP‐LC3/RFP‐LC3 fluorescence intensity ratio was obtained from the confocal images shown in Figure 1, panel E (*n* = 30 cells per experimental condition) and the statistically significant differences were determined by Mann–Whitney (****p < 0.0001). S, grown under starvation conditions; Sel, Selinexor treatment. (B) The BJ fibroblasts harboring either an empty vector or a vector expressing progerin were transduced with the autophagy probe GFP‐LC3‐RFP‐LC3ΔG to measure autophagic flux. Fibroblasts were grown for 12 h in complete media under conditions of starvation (MEM without bovine serum), and thereafter, the cells were treated with 60 nM Selinexor (SEL) or the vehicle alone for 6 days. Subsequently, the fibroblasts were subjected to flow cytometry analysis, and the ratio of GFP‐LC3/RFP‐LC3 was estimated from three independent experiments (> 10,000 cells per experiment), with significant differences determined by two way ANOVA (****p < 0.0001).
**Figure S4:** RNA‐seq analysis of the contrast between WT and HGPS‐1 fibroblasts. (A) Principal components analysis (PCA) of the contrast between WT and HGPS‐1 fibroblasts. The plot spans the samples in a two‐dimensional space, thereby illustrating the clustering of biological replicates and the distances between WT and HGPS‐1 fibroblasts. (B) Volvano Plot visualizing differentially expressed genes in HGPS‐1 versus WT fibroblasts. Blue dots indicate down‐regulated genes, red dots indicate up‐regulated genes, and gray dots indicate unchanged genes. (C) The heat map illustrates the transcriptome profile of WT and HGPS‐1 cells and the hierarchical clustering of differentially expressed transcripts.
**Figure S5:** The analysis of differentially expressed genes (DEGs) in the comparison between WT and HGPS‐1 cells predicts altered autophagy flux in HGPS fibroblasts. (A) Bar plot displaying the percentages of DEGs that were upregulated, downregulated, or remained unchanged in HGPS‐1 fibroblasts in comparison to WT cells. (B) The DEGs related to autophagy are listed. (C) Scheme illustrating the integrative analysis of DEGs using Pathview version 1.5, which predicts impairment in the autophagy‐lysosome axis in HGPS cells.
**Figure S6:** Lysosomal permeabilization in HGPS‐1 fibroblasts. (A) WT cells grown in wells with glass bottom, were incubated with 3 μg/mL of AO (Acridine Orange) for 15 min, after, the cells were incubated at different times (0, 5, 30 and 60 min) with 1 mM of LLoMe (L‐Leucyl‐L‐Leucine methyl ester hydrobromide), to induce lysosomal permeabilization, thus, being a positive control. (B) WT and HGPS‐1 cells grown in wells with glass bottom and were incubated with 3 μg/mL of AO (Acridine Orange) for 15 min prior were analyzed for live imaging in a chamber with temperature control at 37°C, 5% of CO_2_ and humidity control, then were submitted to CLSM (confocal laser scanning microscopy) from Nikon. Representative images of confocal microscopy from three separate experiments are shown. Right. The Pearson correlation Coefficient was calculated (*n* = 30 cells per experimental condition, from three biological replicates) using NIS elements software with significant differences determined by Mann–Whitney (****p < 0.0001).
**Figure S7:** Altered localization and expression of STX17 and LAMP1 proteins in HGPS‐1 fibroblasts. (A and B) WT and HGPS‐1/‐2 fibroblasts grown on coverslips were immunolabeled for STX17 (A) and LAMP1 (B), and counterstained with DAPI to decorate the nuclei. Representative images of confocal microscopy from three separate experiments are shown Right. The fluorescence intensity of STX17 (A) and LAMP1 (B) were quantified (*n* = 100 cells per experimental condition) using NIS elements software (Nikon), with significant differences determined by Mann–Whitney (****p < 0.0001). (C) Lysates from WT and HGPS‐1/‐2 cell cultures were examined by Western blotting using primary antibodies against STX17, LAMP1 or β‐actin (loading control). Typical immunoblots from three independent experiments are shown. Right. The relative levels of STX17 and LAMP1 were estimated, with significant differences determined by unpaired *t* test (*p < 0.0267).
**Figure S8:** Effect of inducible progerin expression on STX17 and LAMP1 proteins in human dermal fibroblasts (HDFs). (A–C) HDFs grown on coverslips were treated with dimethyl sulfoxide (DMSO; vehicle) or doxycycline (Dox) to induce GFP‐Progerin expression. Thereafter, the cells were subjected to immunolabeling for STX17 (A), LAMP1 (B) or SNAP29 (C), counterstained with DAPI to visualize nuclei, and examined using confocal microscopy. *Right*. The fluorescence intensity of LAMP1 (A) and the Fn/c of STX17 and SNAP29 were obtained, with significant differences being determined by Mann–Whitney (****p < 0.0001). (D–F) Lysates from HDF treated with Dox as per A were subjected to western blotting to evaluate GFP‐Progerin, as well as LAMP1 (D), STX17 (E) and SNAP29 (F). Typical immunoblots are shown. *Bottom graphs*. The relative levels of LAMP1 (D), STX17 (E) and SNAP29 (F). were assessed from three independent experiments, using β‐actin as loading control. No statistically significant differences determined by unpaired *t* test.
**Figure S9:** Effect of Selinexor treatment for 3 days on autophagosome maturation in HGPS‐1 fibroblasts. HGPS‐1 fibroblasts were treated with 60 nM Selinexor for three days, and on the second day of treatment, the cells were incubated with CQ for 24 h and then transferred to KRB medium for 60 min (starvation), prior to being subjected to immunostaining for LC3 and STX17 and colocalization analysis. Typical images from two biological replicates are shown. *Bottom*. The Pearson correlation coefficient (PCC) for the STX17‐LC3 colocalization analysis was obtained from two independent experiments (*n* = 20 cells per experimental condition) using NIS Elements software (Nikon), with significant differences determined by *u* Mann–Whitney (*p = 0.0259, ***p = 0.0002).
**Figure S10:** Effect of Selinexor treatment for 3 days on autophagosome‐lysosome fusion in HGPS‐1 fibroblasts. HGPS‐1 fibroblasts were treated with 60 nM Selinexor or the vehicle alone for three days, and on the second day of treatment, the cells were incubated with 100 nM BafA1 for 24 h and then transferred to KRB medium for 60 min (starvation), prior to undergoing immunostaining for colocalizing LC3 and LAMP1. The nuclei were stained with DAPI to enable their visualization. Representative images from two biological replicates are shown. *Right*. The quantity of LAMP1‐positive LC3 puncta was determined from two separate experiments (*n* = 20 cells per experiment), with statistically significative differences being calculated by Mann–Whitney (****p < 0.0001).
**Figure S11:** Viability of WT and HGPS‐1 fibroblasts under Selinexor treatments. (A and B) WT and HGPS‐1 fibroblasts were treated with Selinexor for 3 days (A) or 6 days (B), prior to be subjected to double staining with Anexin V (AV) and Propidium iodide (PI) and further flow cytometry analysis. Data from three biological replicates were obtained as % of live cells (−AV, −PI), % in early apoptosis (+AV, −PI), % in late apoptosis (+AV, +PI) and % in necrosis (AV, +PI), Statistically significant differences were calculated by Two Way Anova (*p < 0.0472, **p < 0.0039, ***p < 0.0004, ****p < 0.0001).
**Figure S12:** Selinexor treatment elicits the nuclear accumulation of the CRM1 target proteins FOXO3 and p53 in WT and HGPS‐1 fibroblasts. (A) WT and HGPS fibroblasts were grown on coverslips and treated with 60 μM Selinexor or the vehicle for three days, prior to being subjected to immunofluorescence analysis using anti‐FOXO3 primary antibodies. Counterstaining with DAPI was used to decorate the nuclei. Typical images from three independent experiments are shown. *Right*. The nuclear to cytoplasmic ratio of fluorescence (Fn/c) of FOXO3 was obtained, with significant differences being determined by Mann–Whitney (****p < 0.0001). (B) HGPS‐1 fibroblast cultures were grown to 70% confluency and treated then with 60 μM Selinexor or the vehicle for three or six days. The fibroblast lysates were obtained and subjected to western blotting using primary antibodies directed to CRM1, p53 and β‐actin (loading control).
**Figure S13:** The decline of pS6 levels in response to starvation is unaltered by Selinexor treatment in HGPS‐1 fibroblasts. (A) Lysates from WT and HGPS‐1 cultures cultured in KRB medium for 30 min (starvation), were subjected to western blotting analysis using antibodies specific to pS6, S6 and β‐actin (loading control) Typical immunoblots from two independent experiments are shown. (B) Lysates from HGPS‐1 fibroblasts that were treated with 60 nM of Selinexor or the vehicle alone for 6 days and then transferred to KRB medium for 30 min (starvation), were subjected to western blotting analysis using antibodies specific to pS6, S6 and β‐actin (loading control). Typical immunoblots from two independent experiments are shown.
**Figure S14:** Cell viability in HGPS fibroblasts by CQ treatments. (A and B) WT and HGPS‐1 fibroblasts were treated with CQ either 12 h (A) or 48 h (B), prior double staining for Anexin V (AV) and Propidium iodide (PI), then flow cytometry analysis was conducted from three biological replicates to obtain: % of live cells (−AV, −PI), % in early apoptosis (+AV, −PI), % in late apoptosis (+AV, +PI) and, % in necrosis (AV, +PI) with statistically significative differences being calculated by two way anova (*p < 0.0495, **p < 0.0034, ***p < 0.0008, ****p < 0.0001).

## Data Availability

The data that support the findings of this study are available from the corresponding author upon reasonable request.
