# Supplementary material for: Progerin Hinders Autophagy Flux at Its Final Stages in Hutchinson‐Gilford Progeria Syndrome Cells, Preventing Its Own Autophagic Degradation
Source: Aging Cell. 2026 Jul 29;25(8):e70649. doi: 10.1111/acel.70649 (PMC13418617; doi:10.1111/acel.70649)
Supplement: Supplementary file 1 — Figure S1: A delay in the formation of autophagosomes in HGPS‐1 fibroblasts. (A and B) WT and HGPS‐1 fibroblasts grown on coverslips were treated with 50 μM CQ for 12 h prior to immunofluorescence staining for LC3 (A) and p62 (B). The fibroblasts were counterstained with DAPI to decorate the nuclei. Representative images of confocal microscopy from three separate experiments are shown. Bottom. The number of LC3 (A) and p62 (B) puncta per cell were quantified (n = 30 cells per experimental condition) using NIS elements software (Nikon), with significant differences determined by Mann–Whitney (****p < 0.0001). Figure S2: Accumulation of SQSTM1/p62 protein in WT and HGPS fibroblasts upon CQ‐mediated autophagy inhibition. (A) WT and HGPS‐1/‐2 fibroblasts grown on coverslips were treated with 50 μM CQ or the vehicle alone for 48 h and then double immunostained for LC3 and p62. Nuclei were labeled with DAPI for visualization prior to confocal microscopy analysis. (B) Lysates from WT and HGPS‐1/HGPS‐2 cultures treated with CQ as per A were subjected to western blotting analysis using antibodies specific to p62 and β‐actin (loading control). Figure S3: Quantitative analysis of the autophagy flux probe GFP‐LC3‐RFP‐LC3ΔG in BJ fibroblasts expressing the empty vector or progerin. (A) The GFP‐LC3/RFP‐LC3 fluorescence intensity ratio was obtained from the confocal images shown in Figure 1, panel E (n = 30 cells per experimental condition) and the statistically significant differences were determined by Mann–Whitney (****p < 0.0001). S, grown under starvation conditions; Sel, Selinexor treatment. (B) The BJ fibroblasts harboring either an empty vector or a vector expressing progerin were transduced with the autophagy probe GFP‐LC3‐RFP‐LC3ΔG to measure autophagic flux. Fibroblasts were grown for 12 h in complete media under conditions of starvation (MEM without bovine serum), and thereafter, the cells were treated with 60 nM Selinexor (SEL) or the vehicle alone for 6 days. Subsequ [file ACEL-25-e70649-s001.docx]

**Supplementary information**

**Progerin hinders autophagy flux at its final stages in Hutchinson-Gilford progeria syndrome cells, preventing its own autophagic degradation**

Ian García-Aguirre^1^, Jesús Alejandro Reyes-Martínez^2^, Juan Unzueta^2^, Francisco Guevara-Namorado^2^, Solangy Lizcano-Meneses^1,3^, Susana Gonzalo^4^, Angel Baldan^4^, Claudia Rangel^5,6^, Gerardo J. Alanis-Funes^7^, Francisco Garcia-Sierra^3^, Kevin Ruiz-Fajardo^2^, Simon Gormes-Pinchanski^1^, Susana Castro-Obregón^8^, Aranza Meza-Dorantes^1^, Rocio Alejandra Chavez-Santoscoy^5^, Isabel Arrieta-Cruz^9^, Paola Tristán-Aburto^1^, Jonathan J. Magaña^1,10*^, Bulmaro Cisneros^2*^.

^1^Departamento de Bioingeniería, Escuela de Ingeniería y Ciencias, Tecnologico de Monterrey, Ciudad de México 14380, Mexico.

^2^Departamento de Genética y Biología Molecular, Centro de Investigación y de Estudios Avanzados, Ciudad de México 07360, México.

^3^Department of Cell Biology, Center for Research and Advanced Studies of the National Polytechnic Institute, Mexico City, Mexico.

^4^Edward A. Doisy Department of Biochemistry and Molecular Biology, Saint Louis University School of Medicine, St. Louis, Missouri, USA.

^5^Escuela de Ingeniería y Ciencias, Tecnológico de Monterrey, Monterrey, Mexico.

^6^Computational and Integrative Biology, National Institute of Genomic Medicine, Periférico Sur 4809 Arenal Tepepan 14610.

^7^School of Engineering and Sciences, Tecnologico de Monterrey, Campus Querétaro, Querétaro 76130, QRO., Mexico.

^8^Instituto de Fisiología Celular, UNAM, Ciudad Universitaria, CP04510 Ciudad de México, México.

^9^Departamento de Investigación Básica, División de Investigación, Instituto Nacional de Geriatría, Secretaría de Salud, Ciudad de México, Mexico.

^10^Laboratorio de Medicina Genómica, Departamento de Genética (CENIAQ), Instituto Nacional de Rehabilitación-Luis Guillermo Ibarra Ibarra (INR-LGII), Ciudad de México 14389, Mexico.

**^*^Corresponding information:**

Bulmaro Cisneros Vega: [bcisnero@cinvestav.mx](mailto:bcisnero@cinvestav.mx)

Jonathan J. Magaña: magana.jj@tec.mx

**Keywords:** Aging, Progeria, Autophagy, Lysosomes

**This file includes:**

- **Supplementary Figure 1.** A delay in the formation of autophagosomes in HGPS-1 fibroblasts.
- **Supplementary Figure 2.** Accumulation of SQSTM1/p62 protein in WT and HGPS fibroblasts upon CQ-mediated autophagy inhibition.
- **Supplementary Figure 3.** Quantitative analysis of the autophagy flux probe GFP-LC3-RFP-LC3ΔG in BJ fibroblasts expressing the empty vector or progerin.
- **Supplementary Figure 4.** RNA-seq analysis of the contrast between WT and HGPS-1 fibroblasts.
- **Supplementary Figure 5.** The analysis of differentially expressed genes (DEGs) in the comparison between WT and HGPS-1 cells predicts altered autophagy flux in HGPS fibroblasts.
- **Supplementary Figure 6.** Lysosomal permeabilization in HGPS-1 fibroblasts.
- **Supplementary Figure 7.** Altered localization and expression of STX17 and LAMP1 proteins in HGPS fibroblasts.
- **Supplementary Figure 8.** Effect of inducible progerin expression on STX17 and LAMP1 proteins in human dermal fibroblasts (HDFs).
- **Supplementary Figure 9.** Effect of Selinexor treatment for 3 days on autophagosome maturation in HGPS-1 fibroblasts.
- **Supplementary Figure 10.** Effect of Selinexor treatment for 3 days on autophagosome-lysosome fusion in HGPS-1 fibroblasts.
- **Supplementary Figure 11.** Viability of WT and HGPS-1 fibroblasts under Selinexor treatments.
- **Supplementary Figure 12.** Selinexor treatment elicits the nuclear accumulation of the CRM1 target proteins FOXO3 and p53 in WT and HGPS-1 fibroblasts.
- **Supplementary Figure 13.** The decline of pS6 levels in response to starvation is unaltered by Selinexor treatment in HGPS-1 fibroblasts.
- **Supplementary Figure 14.** Cell viability in HGPS fibroblasts by CQ treatments.
- **Long versions of legends from main figure 1 and 2.**
- **Materials and methods.**

**
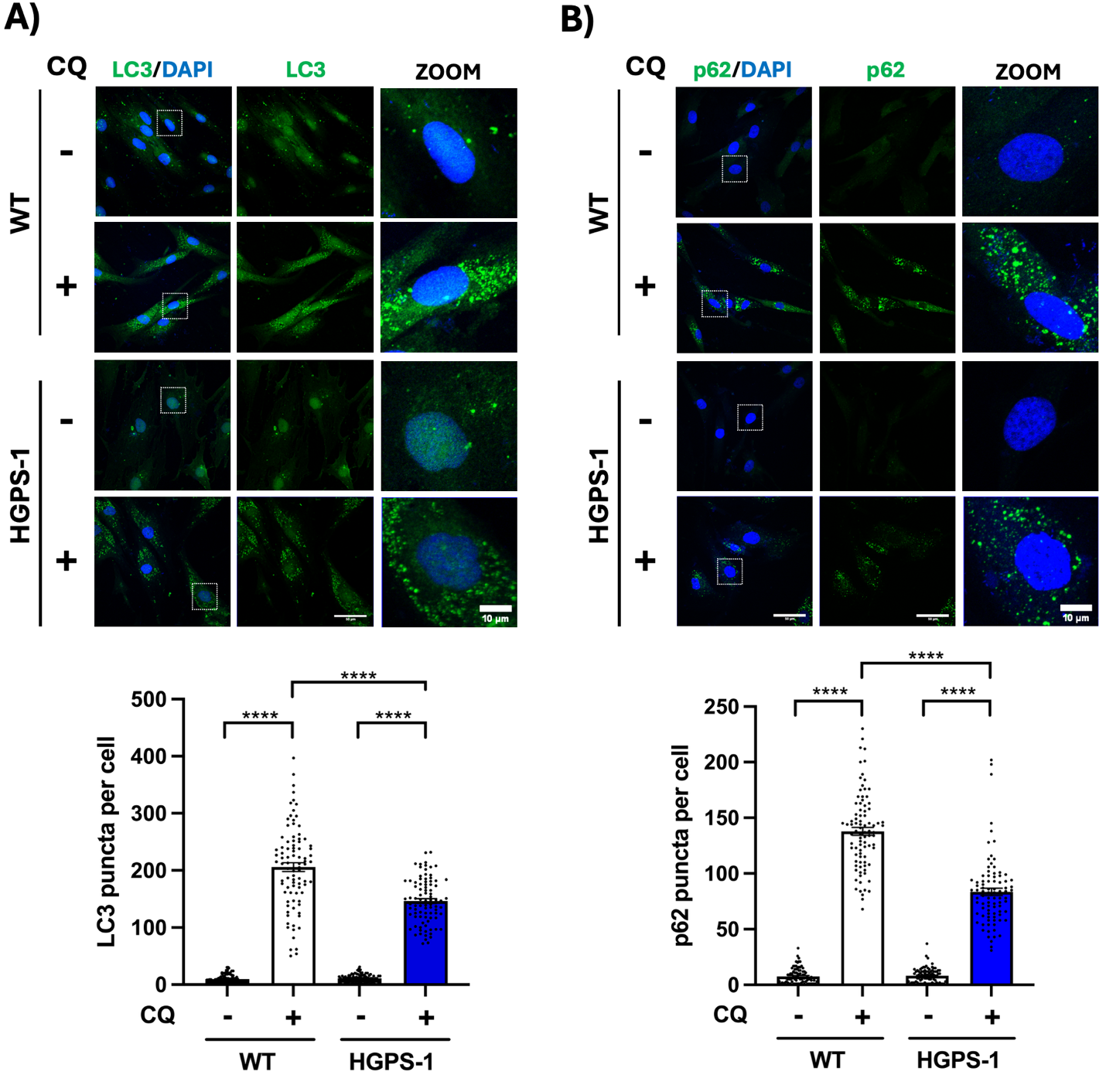
**

**Supplementary Figure 1.** **A delay in the formation of autophagosomes in HGPS-1 fibroblasts.** (A-B) WT and HGPS-1 fibroblasts grown on coverslips were treated with 50 μM CQ for 12 h prior to immunofluorescence staining for LC3 (A) and p62 (B). The fibroblasts were counterstained with DAPI to decorate the nuclei. Representative images of confocal microscopy from 3 separate experiments are shown. ***Bottom.*** The number of LC3 (A) and p62 (B) puncta per cell were quantified (n= 30 cells per experimental condition) using NIS elements software (Nikon), with significant differences determined by Mann-Whitney (**** *p*< 0.0001).

**
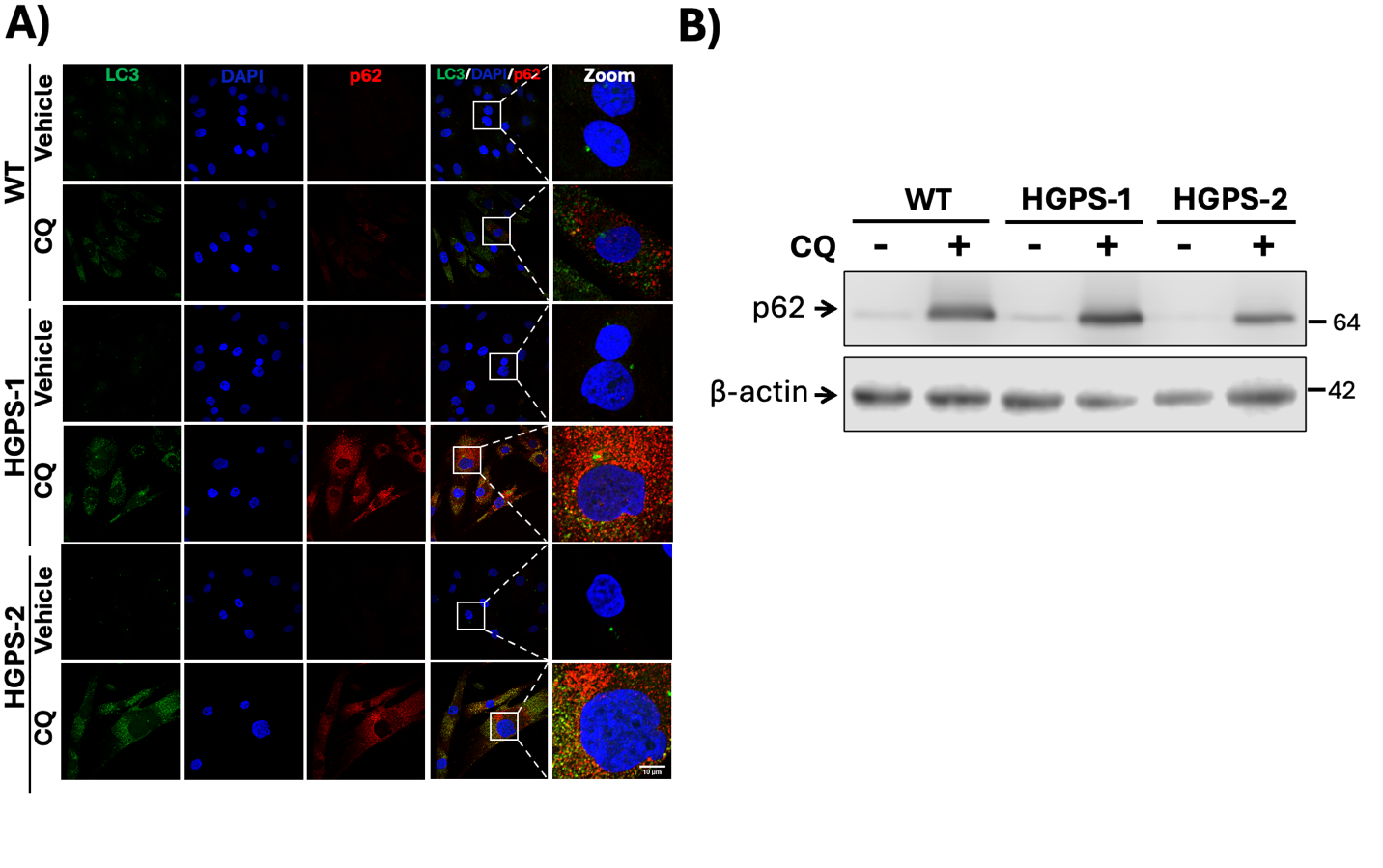
**

**Supplementary Figure 2. Accumulation of SQSTM1/p62 protein in WT and HGPS fibroblasts upon CQ-mediated autophagy inhibition.** (A) WT and HGPS-1/-2 fibroblasts grown on coverslips were treated with 50 μM CQ or the vehicle alone for 48 h and then double immunostained for LC3 and p62. Nuclei were labeled with DAPI for visualization prior to confocal microscopy analysis. (B) Lysates from WT and HGPS-1/HGPS-2 cultures treated with CQ as per A were subjected to western blotting analysis using antibodies specific to p62 and β-actin (loading control).

**
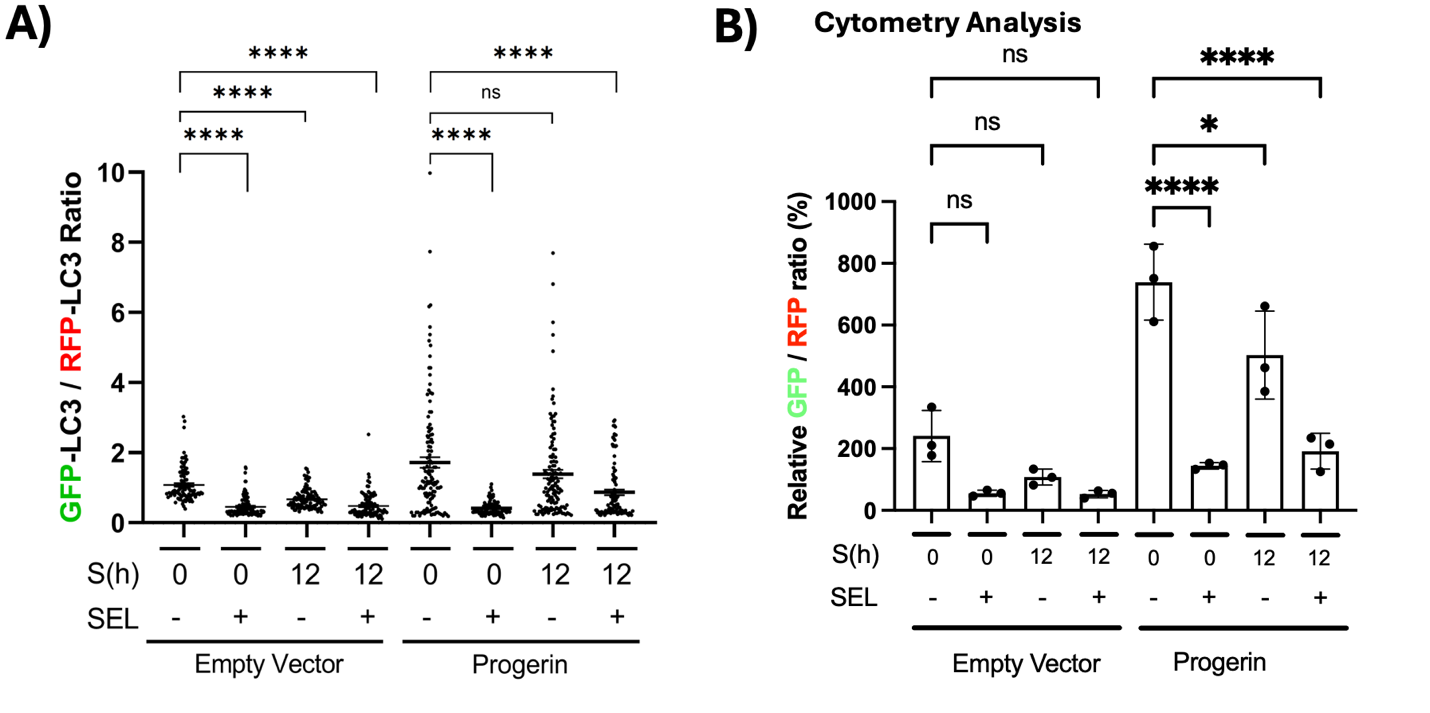
**

**Supplementary Figure 3. Quantitative analysis of the autophagy flux probe GFP-LC3-RFP-LC3ΔG in BJ fibroblasts expressing the empty vector or progerin.** (A) The GFP-LC3/RFP-LC3 fluorescence intensity ratio was obtained from the confocal images shown in Figure 1, panel E (n = 30 cells per experimental condition) and the statistically significant differences were determined by Mann-Whitney (**** p< 0.0001). S, grown under starvation conditions; Sel, Selinexor treatment. (B) The BJ fibroblasts harboring either an empty vector or a vector expressing progerin were transduced with the autophagy probe GFP-LC3-RFP-LC3ΔG to measure autophagic flux. Fibroblasts were grown for 12 h in complete media under conditions of starvation (MEM without bovine serum), and thereafter, the cells were treated with 60 nM Selinexor (SEL) or the vehicle alone for 6 days. Subsequently, the fibroblasts were subjected to flow cytometry analysis, and the ratio of GFP-LC3/RFP-LC3 was estimated from three independent experiments (>10,000 cells per experiment), with significant differences determined by 2way ANOVA (****p< 0.0001).


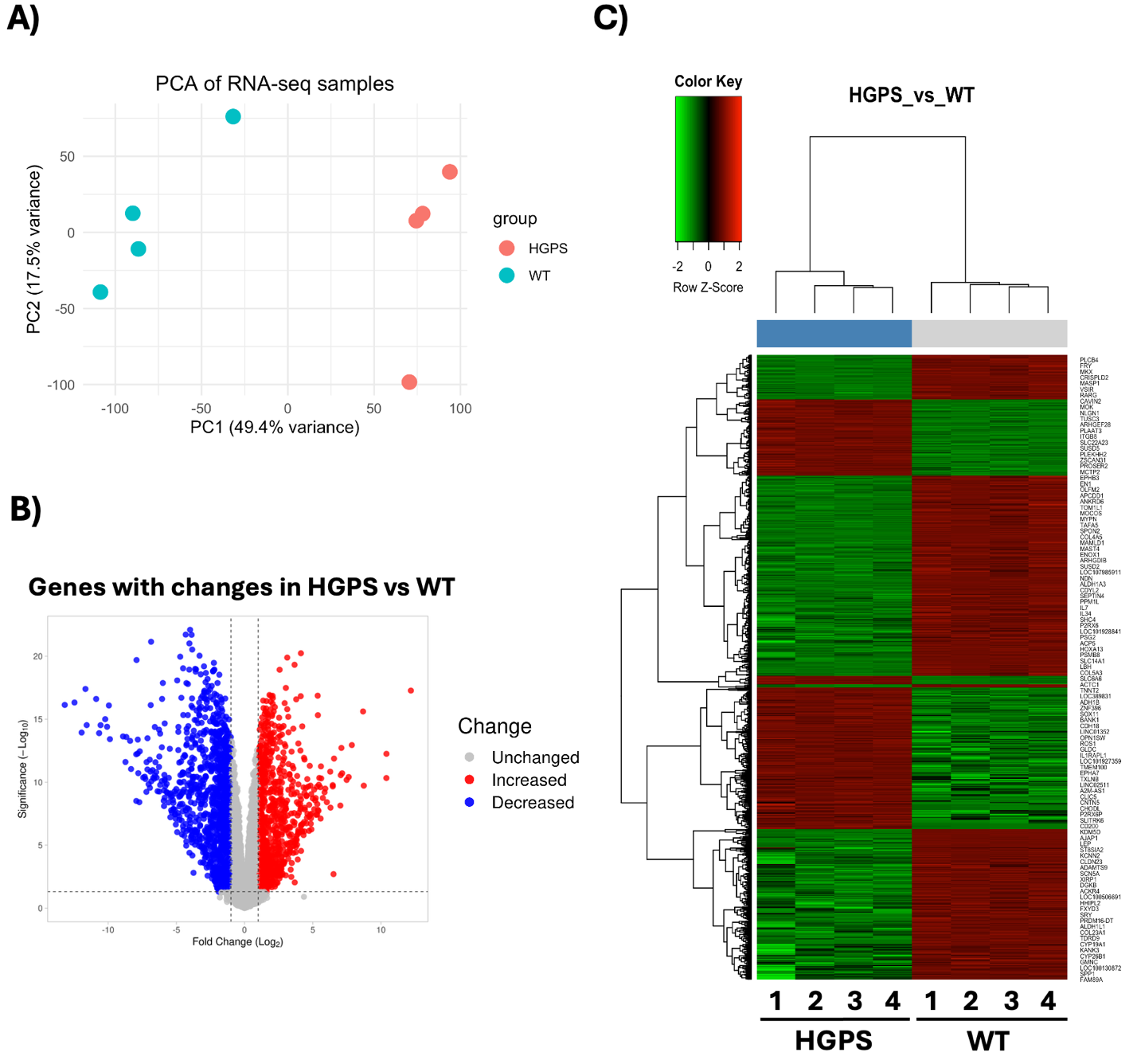


**Supplementary Figure 4. RNA-seq analysis of the contrast between WT and HGPS-1 fibroblasts.** (A) Principal components analysis (PCA) of the contrast between WT and HGPS-1 fibroblasts. The plot spans the samples in a two-dimensional space, thereby illustrating the clustering of biological replicates and the distances between WT and HGPS-1 fibroblasts. (B) Volvano Plot visualizing differentially expressed genes in HGPS-1 versus WT fibroblasts. Blue dots indicate down-regulated genes, red dots indicate up-regulated genes, and gray dots indicate unchanged genes. (C) The heat map illustrates the transcriptome profile of WT and HGPS-1 cells and the hierarchical clustering of differentially expressed transcripts.


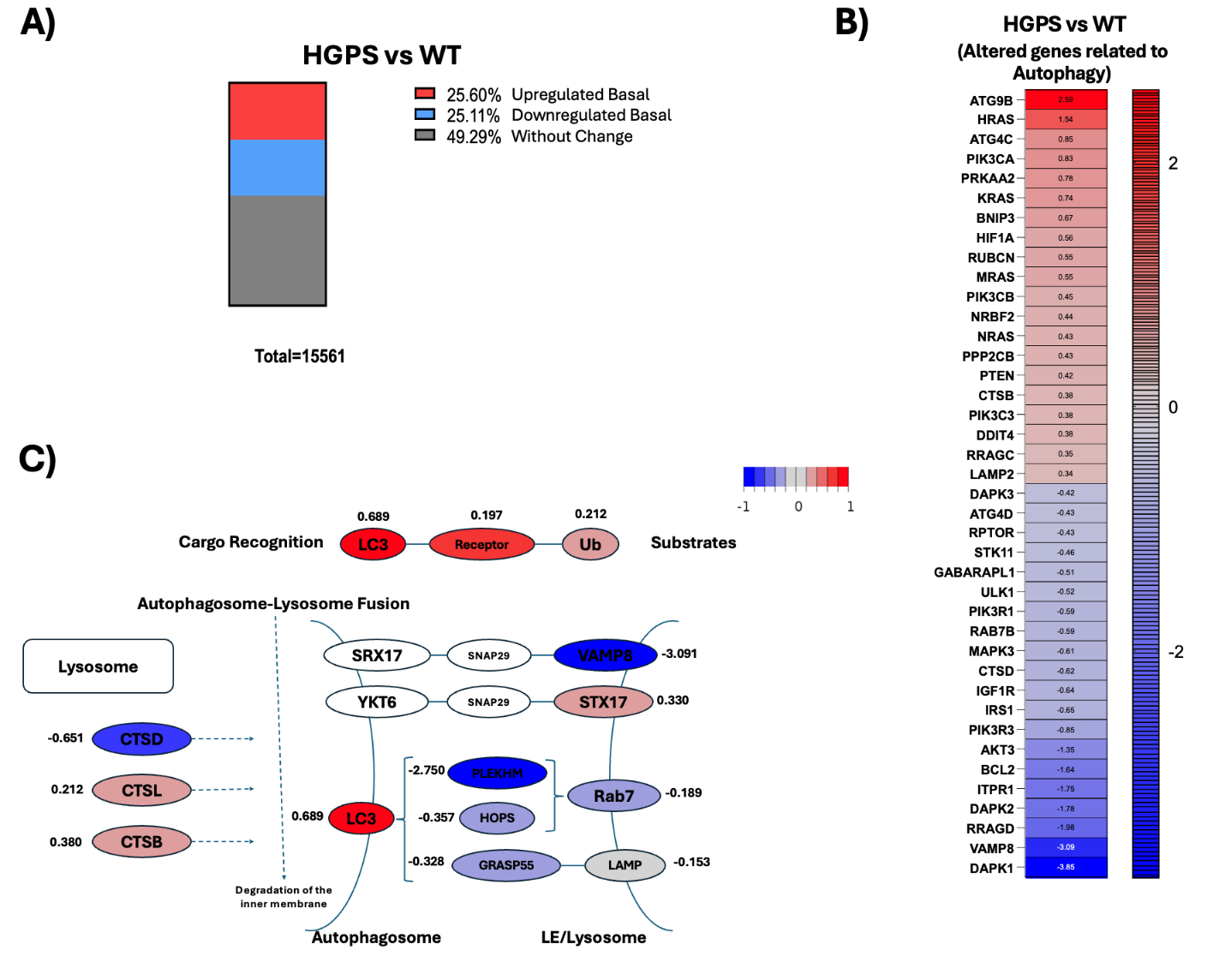


**Supplementary Figure 5. The analysis of differentially expressed genes (DEGs) in the comparison between WT and HGPS-1 cells predicts altered autophagy flux in HGPS fibroblasts.** A) Bar plot displaying the percentages of DEGs that were upregulated, downregulated, or remained unchanged in HGPS-1 fibroblasts in comparison to WT cells. B) The DEGs related to autophagy are listed. C) Scheme illustrating the integrative analysis of DEGs using Pathview version 1.5, which predicts impairment in the autophagy-lysosome axis in HGPS cells.


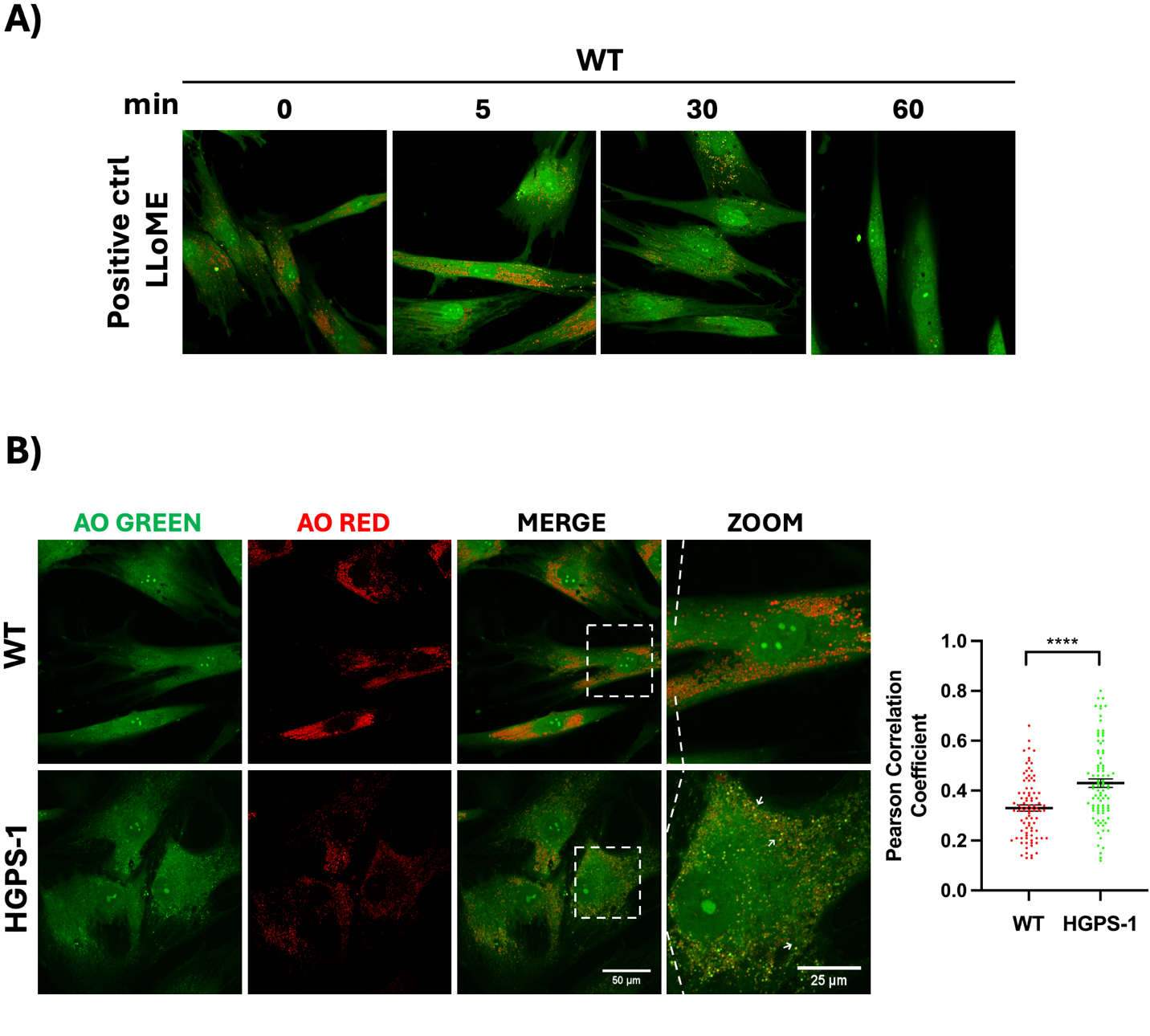


**Supplementary Figure 6. Lysosomal permeabilization in HGPS-1 fibroblasts.** A) WT cells grown in wells with glass bottom, were incubated with 3μg/ml of AO (Acridine Orange) for 15 min, after, the cells were incubated at different times (0, 5, 30 and 60 min) with 1mM of LLoMe (L-Leucyl-L-Leucine methyl ester hydrobromide), to induce lysosomal permeabilization, thus, being a positive control. B) WT and HGPS-1 cells grown in wells with glass bottom and were incubated with 3μg/ml of AO (Acridine Orange) for 15 min prior were analyzed for live imaging in a chamber with temperature control at 37°C, 5% of C0_2_ and humidity control, then were submitted to CLSM (confocal laser scanning microscopy) from Nikon. Representative images of confocal microscopy from 3 separate experiments are shown. ***Right.*** The Pearson correlation Coefficient was calculated (n= 30 cells per experimental condition, from three biological replicates) using NIS elements software with significant differences determined by Mann-Whitney (**** *p*< 0.0001).


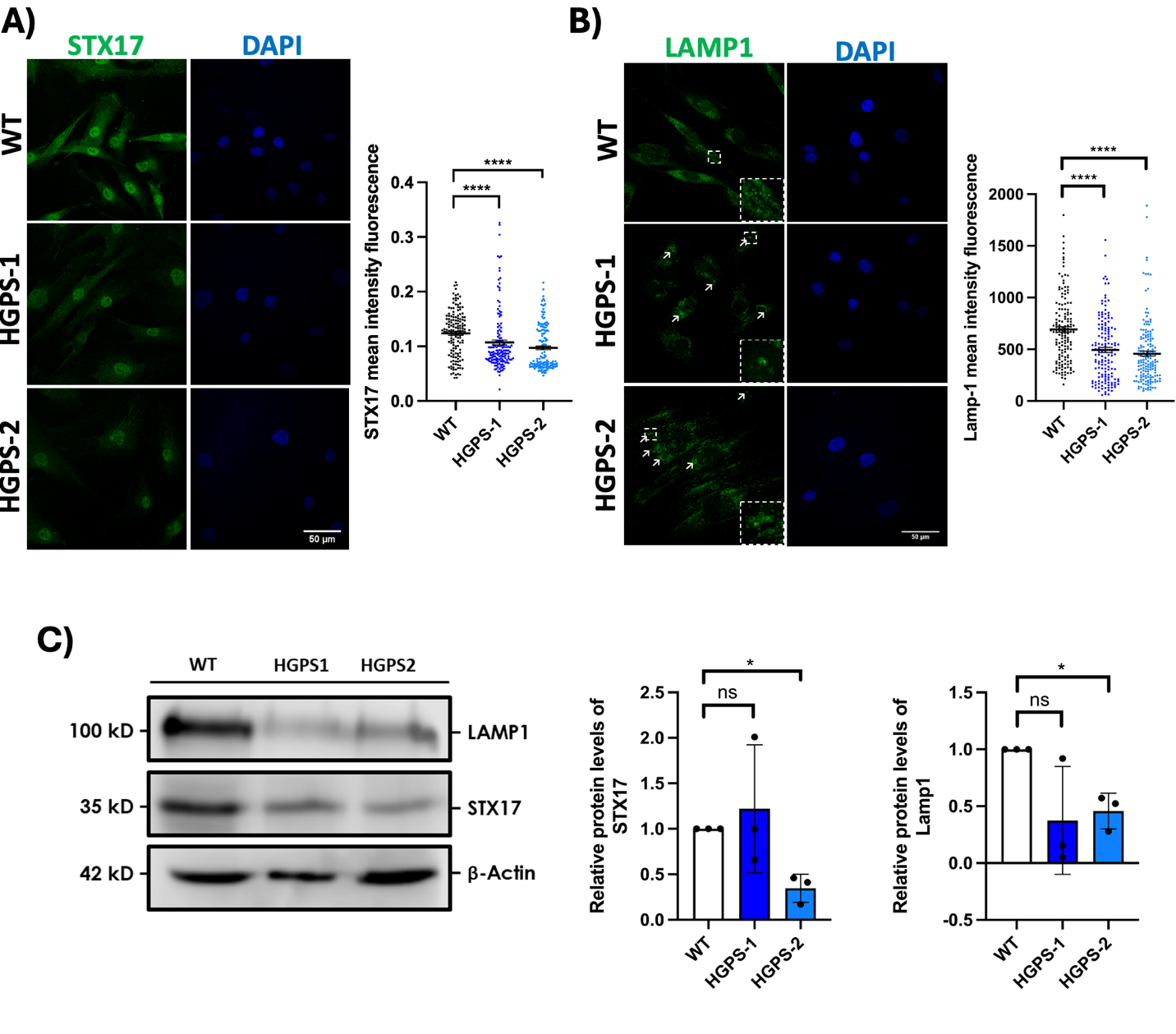


**Supplementary Figure 7.** **Altered localization and expression of STX17 and LAMP1 proteins in HGPS-1 fibroblasts.** A-B) WT and HGPS-1/-2 fibroblasts grown on coverslips were immunolabeled for STX17 (A) and LAMP1 (B), and counterstained with DAPI to decorate the nuclei. Representative images of confocal microscopy from 3 separate experiments are shown ***Right.*** The fluorescence intensity of STX17 (A) and LAMP1 (B) were quantified (n= 100 cells per experimental condition) using NIS elements software (Nikon), with significant differences determined by Mann-Whitney (**** *p*< 0.0001). (C) Lysates from WT and HGPS-1/-2 cell cultures were examined by Western blotting using primary antibodies against STX17, LAMP1 or β-actin (loading control). Typical immunoblots from three independent experiments are shown. ***Right.*** The relative levels of STX17 and LAMP1 were estimated, with significant differences determined by unpaired *t* test (* *p*< 0.0267).


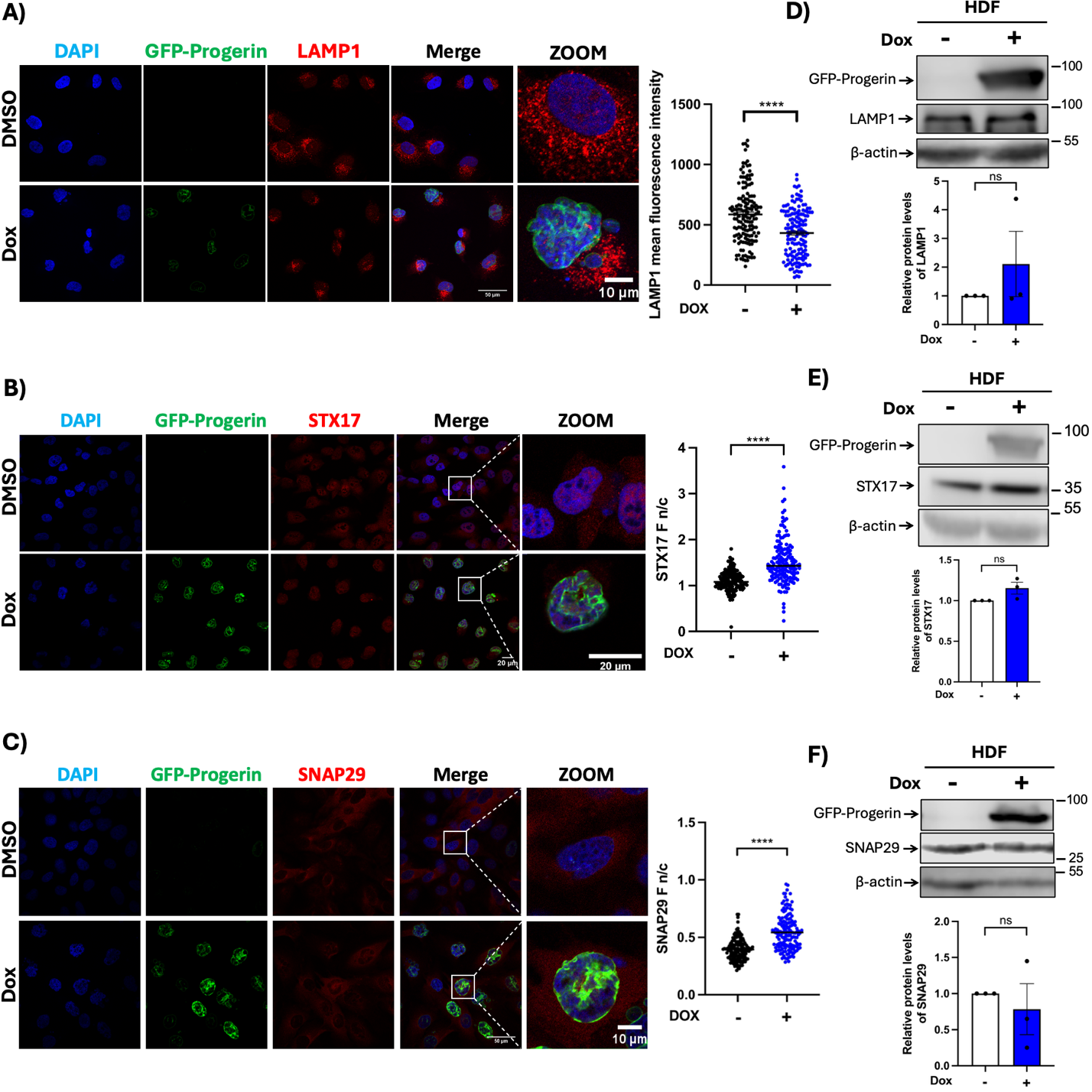


**Supplementary Figure 8.** **Effect of inducible progerin expression on STX17 and LAMP1 proteins in human dermal fibroblasts (HDFs).** (A-C) HDFs grown on coverslips were treated with dimethyl sulfoxide (DMSO; vehicle) or doxycycline (Dox) to induce GFP-Progerin expression. Thereafter, the cells were subjected to immunolabeling for STX17 (A), LAMP1 (B) or SNAP29 (C), counterstained with DAPI to visualize nuclei, and examined using confocal microscopy. *Right*. The fluorescence intensity of LAMP1 (A) and the Fn/c of STX17 and SNAP29 were obtained, with significant differences being determined by Mann-Whitney (**** *p*< 0.0001). (D-F) Lysates from HDF treated with Dox as per A were subjected to western blotting to evaluate GFP-Progerin, as well as LAMP1 (D), STX17 (E) and SNAP29 (F). Typical immunoblots are shown. *Bottom graphs.* The relative levels of LAMP1 (D), STX17 (E) and SNAP29 (F). were assessed from three independent experiments, using β-actin as loading control. No statistically significant differences determined by unpaired *t* test.

**
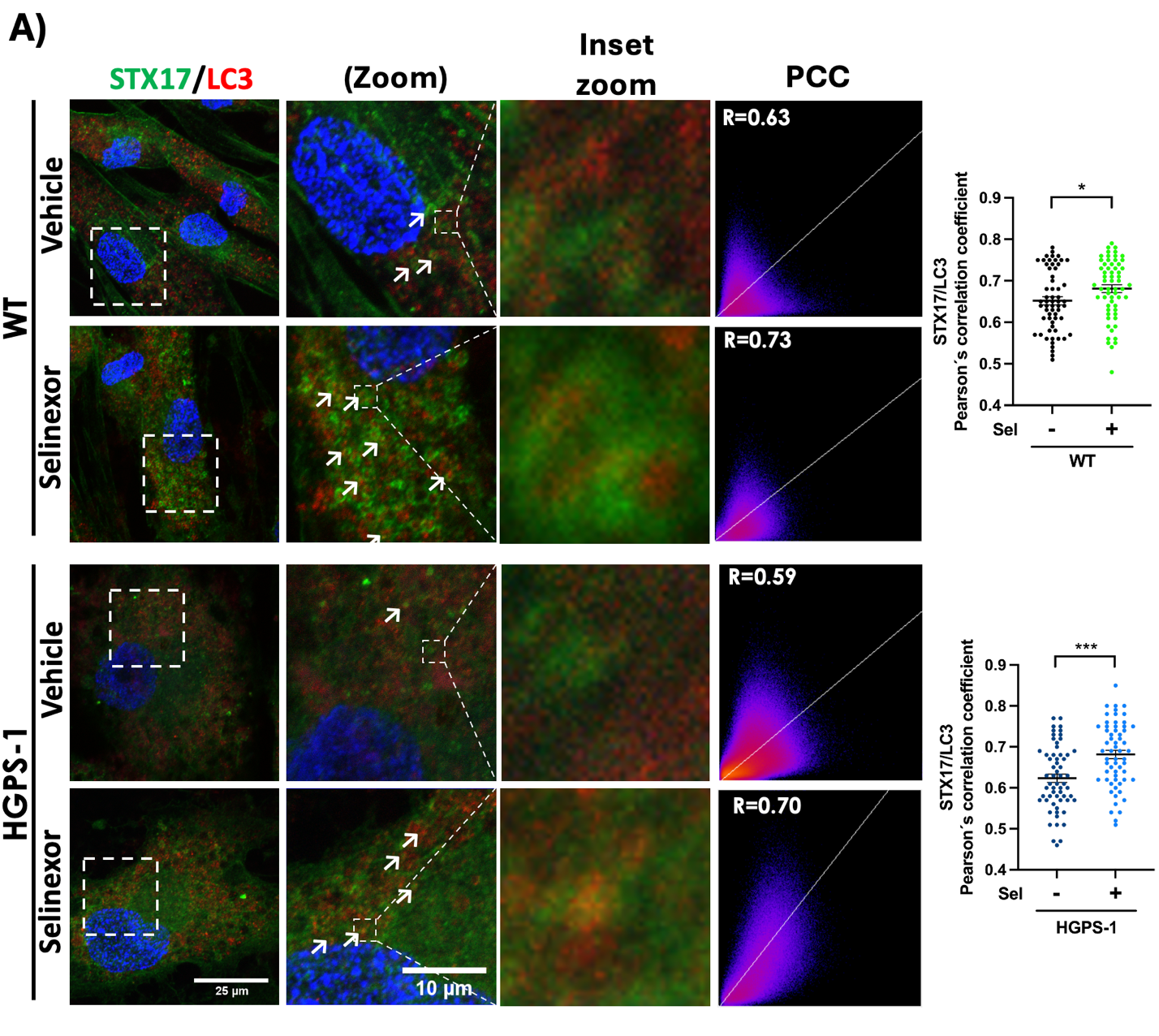
**

**Supplementary Figure 9. Effect of Selinexor treatment for 3 days on autophagosome maturation in HGPS-1 fibroblasts.** HGPS-1 fibroblasts were treated with 60 nM Selinexor for three days, and on the second day of treatment, the cells were incubated with CQ for 24 h and then transferred to KRB medium for 60 minutes (starvation), prior to being subjected to immunostaining for LC3 and STX17 and colocalization analysis. Typical images from two biological replicates are shown. *Bottom.* The Pearson correlation coefficient (PCC) for the STX17-LC3 colocalization analysis was obtained from two independent experiments (n= 20 cells per experimental condition) using NIS Elements software (Nikon), with significant differences determined by u Mann-Whitney (* *p*= 0.0259, *** *p*= 0.0002).

**
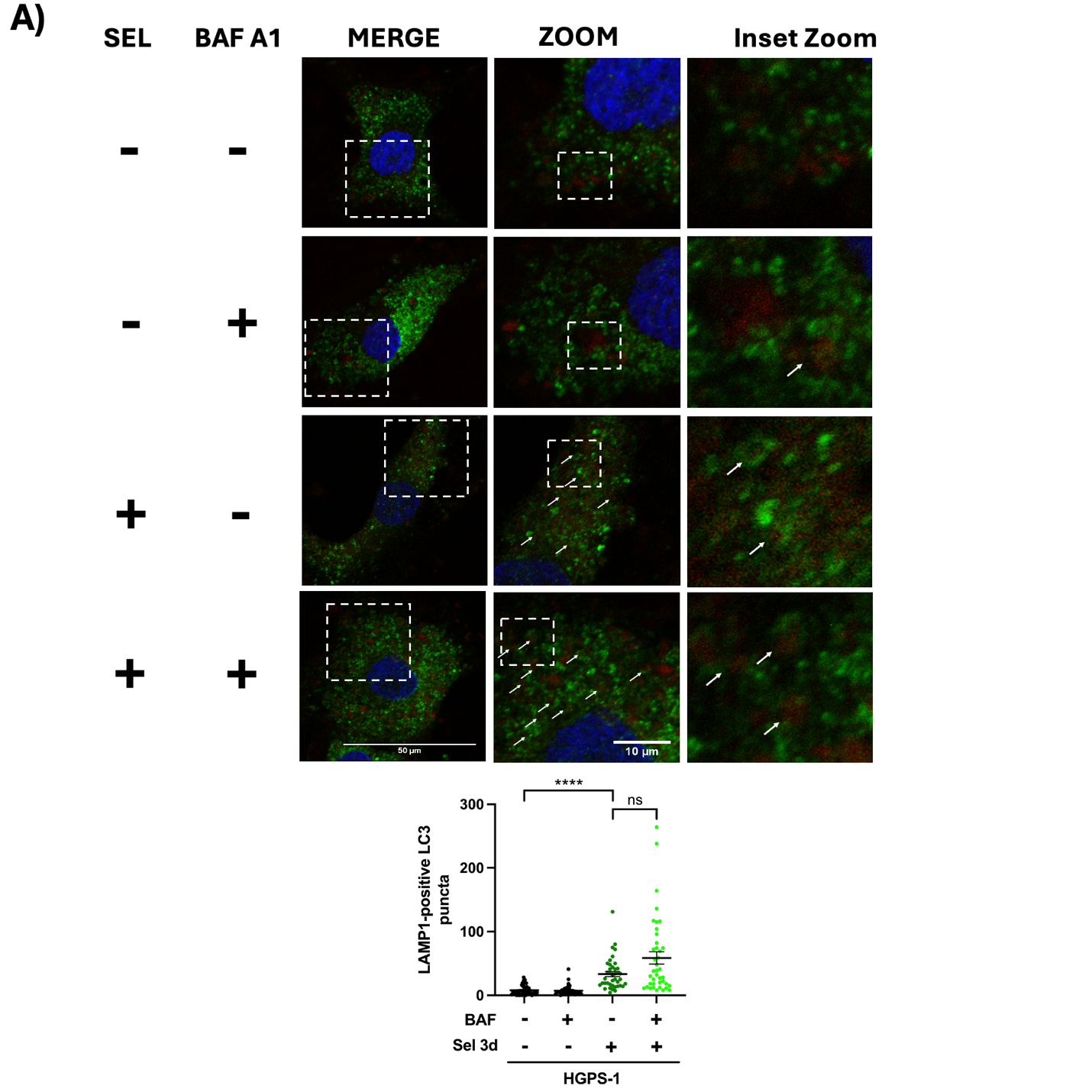
**

**Supplementary Figure 10 Effect of Selinexor treatment for 3 days on autophagosome-lysosome fusion in HGPS-1 fibroblasts.** HGPS-1 fibroblasts were treated with 60 nM Selinexor or the vehicle alone for three days, and on the second day of treatment, the cells were incubated with 100 nM BafA1 for 24 h and then transferred to KRB medium for 60 minutes (starvation), prior to undergoing immunostaining for colocalizing LC3 and LAMP1. The nuclei were stained with DAPI to enable their visualization. Representative images from two biological replicates are shown. *Right.* The quantity of LAMP1-positive LC3 puncta was determined from two separate experiments (n= 20 cells per experiment), with statistically significative differences being calculated by Mann-Whitney (**** *p*< 0.0001).

**
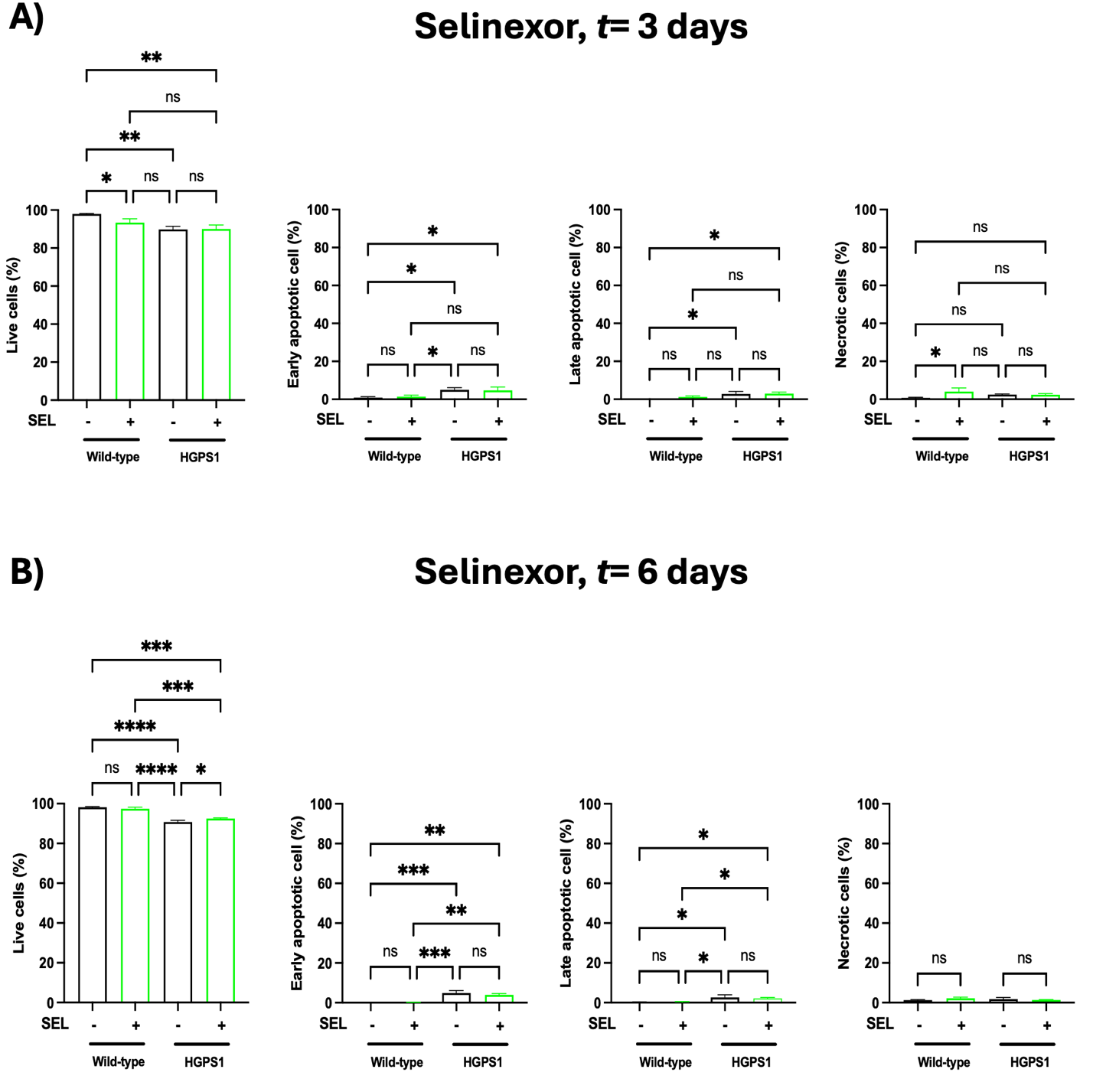
**

**Supplementary Figure 11. Viability of WT and HGPS-1 fibroblasts under Selinexor treatments.** (A-B) WT and HGPS-1 fibroblasts were treated with Selinexor for 3 days (A) or 6 days (B), prior to be subjected to double staining with Anexin V (AV) and Propidium iodide (PI) and further flow cytometry analysis. Data from three biological replicates were obtained as % of live cells (-AV, -PI), % in early apoptosis (+AV, -PI), % in late apoptosis (+AV, +PI) and % in necrosis (AV, +PI), Statistically significant differences were calculated by Two Way Anova (* *p<* 0.0472 , ** *p<* 0.0039, *** *p<* 0.0004, **** *p<* 0.0001).

**
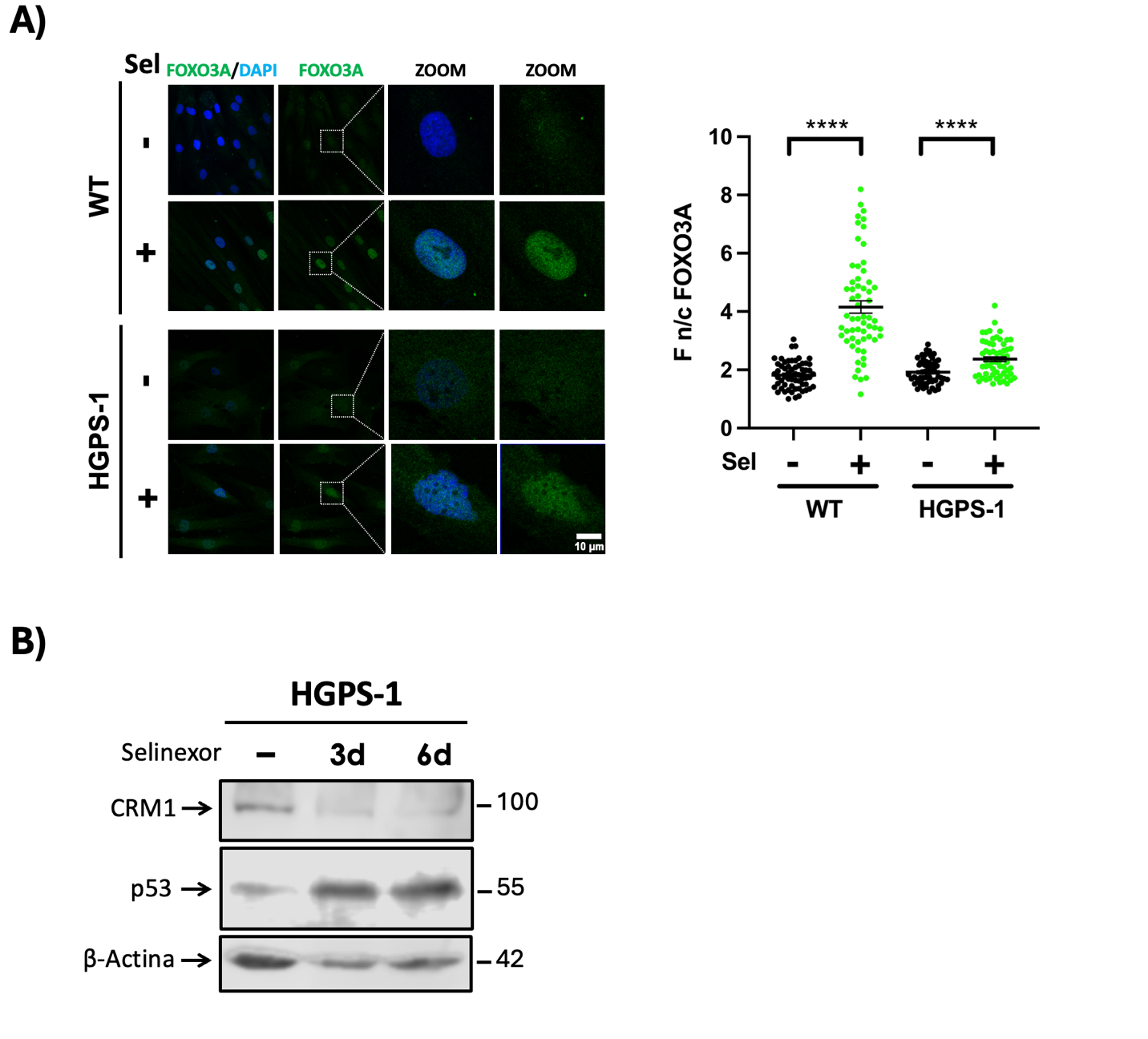
**

**Supplementary Figure 12. Selinexor treatment elicits the nuclear accumulation of the CRM1 target proteins FOXO3 and p53 in WT and HGPS-1 fibroblasts.** (A) WT and HGPS fibroblasts were grown on coverslips and treated with 60 µM Selinexor or the vehicle for three days, prior to being subjected to immunofluorescence analysis using anti-FOXO3 primary antibodies. Counterstaining with DAPI was used to decorate the nuclei. Typical images from three independent experiments are shown. *Right.* The nuclear to cytoplasmic ratio of fluorescence (Fn/c) of FOXO3 was obtained, with significant differences being determined by Mann-Whitney (**** *p*< 0.0001). (B) HGPS-1 fibroblast cultures were grown to 70% confluency and treated then with 60 µM Selinexor or the vehicle for three or six days. The fibroblast lysates were obtained and subjected to western blotting using primary antibodies directed to CRM1, p53 and β-actin (loading control).

**
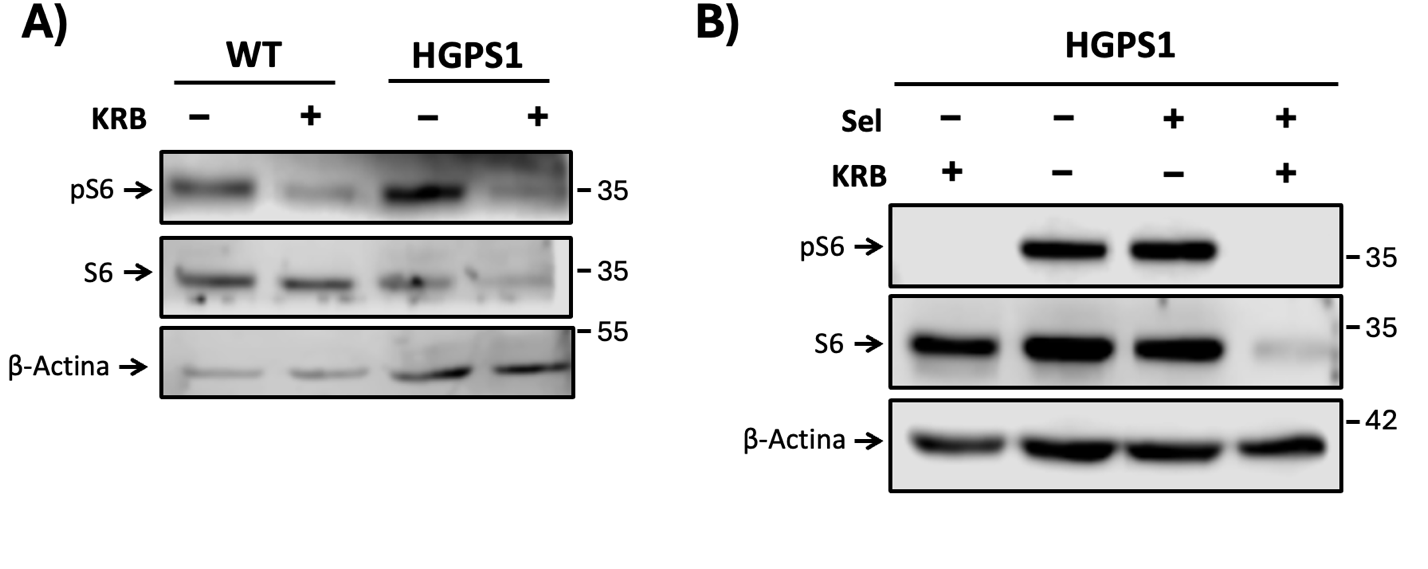
**

**Supplementary Figure 13. The decline of pS6 levels in response to starvation is unaltered by Selinexor treatment in HGPS-1 fibroblasts.** A) Lysates from WT and HGPS-1 cultures cultured in KRB medium for 30 minutes (starvation), were subjected to western blotting analysis using antibodies specific to pS6, S6 and β-actin (loading control) Typical immunoblots from two independent experiments are shown. B) Lysates from HGPS-1 fibroblasts that were treated with 60 nM of Selinexor or the vehicle alone for 6 days and then transferred to KRB medium for 30 minutes (starvation), were subjected to western blotting analysis using antibodies specific to pS6, S6 and β-actin (loading control). Typical immunoblots from two independent experiments are shown.

**
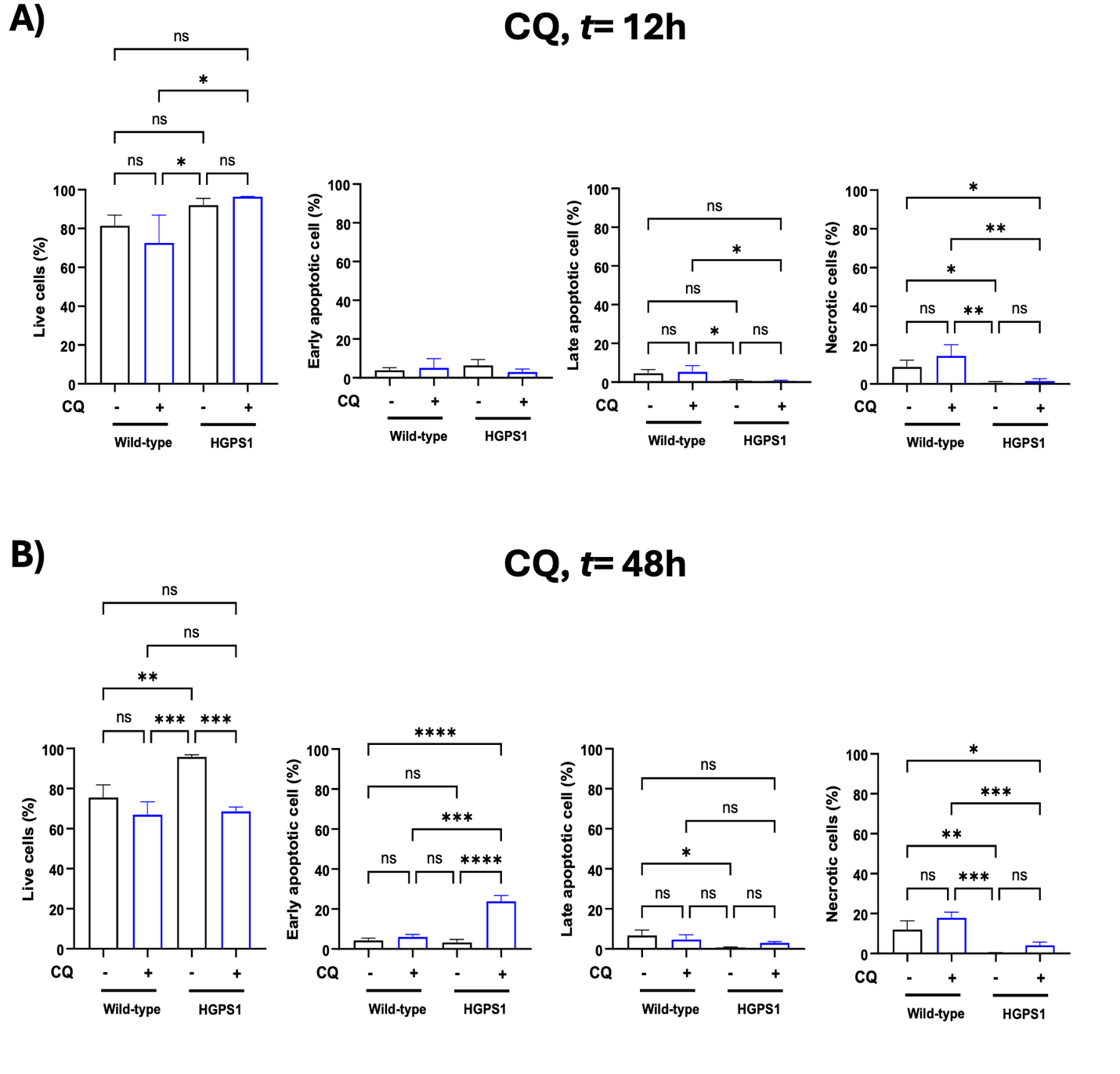
**

**Supplementary Figure S14. Cell viability in HGPS fibroblasts by CQ treatments.** A-B) WT and HGPS-1 fibroblasts were treated with CQ either 12 h (A) or 48 h (B), prior double staining for Anexin V (AV) and Propidium iodide (PI), then flow cytometry analysis was conducted from three biological replicates to obtain: % of live cells (-AV, -PI), % in early apoptosis (+AV, -PI), % in late apoptosis (+AV, +PI) and, % in necrosis (AV, +PI) with statistically significative differences being calculated by two way anova (* *p*< 0.0495, ** *p*< 0.0034, *** *p*< 0.0008, **** *p*< 0.0001).

**Long versions of legends from main figure 1 and 2**

**Figure 1.- Autophagy flux is impaired in HGPS cells, and its restoration via Selinexor treatment promotes the clearance of progerin. (**A) Autophagosomes accumulate in HGPS cells. WT and HGPS-1/-2 fibroblasts were treated with 50 μM CQ or the vehicle alone for 48 h prior to immunofluorescence staining for LC3, and counterstaining with DAPI to visualize nuclei. Typical images from three independent experiments are shown. *Right*. The number of LC3 puncta per cell was determined using NIS elements software (Nikon; > 150 cells per experimental condition), with significant differences determined by Mann-Whitney test (**** *p*< 0.0001). (B) The conversion of LC3-I to LC3-II is increased in HGPS cells. Lysates from WT and HGPS-1/-2 cells previously treated with CQ as per A) were analyzed by immunoblotting using specific antibodies to LC3 and β-actin (loading control). Typical WB images are shown. *Bottom.* The LC3-II/LC3-I ratio was measured from three independent experiments, with significant differences obtained by unpaired t test (* *p*< 0.0489; ** *p*< 0.0066; *****p*< 0.0001). (C**)** WT and HGPS-1/-2 fibroblasts were treated with 50 μM CQ or the vehicle alone for 48 h prior to staining with the Cyto-ID autophagy detection kit and analyzed by flow cytometry. Autophagic vacuoles were estimated from three independent experiments (>10,000 cells per experiment), with significant differences determined by unpaired t-test (** *p*< 0.0066; *****p*< 0.0001) (D**)** Lysosomal turnover of LC3-II is diminished in HGPS cells. Western blot analyses were conducted on lysate samples from WT and HGPS-1 cells cultured in complete medium or KRB medium (a starvation condition) and treated for 4 h with lysosomal protease inhibitors E64 (10 μg/ml) and pepstatin A (10 μg/ml). Antibodies specific to LC3 and β-actin (loading control) were used, and a representative blot is shown***.*** *Bottom****.*** The ratio of LC3II/LC3I within lysosomes was estimated from three independent experiments, with significant differences determined by unpaired t-test (* *p*< 0.0489). (E**)** BJ cells harboring either a control (empty) or a vector expressing progerin were transduced with an autophagy probe GFP-LC3-RFP-LC3ΔG to measure autophagic flux. Cells were grown for 12 h in complete media or under conditions of starvation (MEM without bovine serum) and thereafter, the cells were treated with 60 nM Selinexor or vehicle alone for 6 days. Subsequently, the cells were subjected to confocal microscopy analysis. (F**)** Lysates from BJ cells grown and treated as per E were analyzed by Western blotting using antibodies specific to LC3, GFP, progerin and β-actin (loading control). A representative blot of three independent experiments is shown.

**Figure 2.- Impediment of autophagy in late stages in HGPS cells and its release by Selinexor treatment. (**A) The top ten Gene Ontology biological processes that were found to be enriched in HGPS cells by RNA sequencing include Lysosome and Autophagy functions. (B-C) The differentially expressed genes in HGPS cells that are targeted by TFEB (B) or associated with lysosomal function (C) are displayed. (D) The TFEB phosphorylation state was analyzed by Western blotting in lysate samples from WT and HGPS-1 cells grown in KRB medium for 4 h. Antibodies specific to TFEB, progerin or β-actin (loading control) were used, and a representative blot from three separate experiments is shown. (E) KRB-mediated starvation induces the nuclear accumulation of TFEB. WT and HGPS-1 cells were cultured in KRB medium for 4 h, prior to be immunostaining with TFEB primary antibodies and counterstaining with DAPI to decorate the nuclei. Typical confocal images from three independent experiments are shown. (F) WT and HGPS-1/-2 fibroblasts grown on coverslips were stained with Lysobrite and Hoechst to decorate lysosomes and nuclei respectively, prior to being subjected to confocal microscopy analysis. Typical images from three different biological replicates are shown. (G**)** The number of lysosomes per cell and per area (bottom) were quantified (n= 100 cells per experimental condition) and significant differences were determined by Mann-Whitney (**** *p*< 0.0001). (H) The lysosomal intracellular activity was assessed in WT and HGPS-1/-2 fibroblasts by staining the cells with a Lysosomal Intracellular Activity Kit and further analysis by flow cytometry. WT cells treated with 200 nM Bafilomycin A1 for 24 were utilized as a negative control. Statistically significant differences were determined from three independent experiments by unpaired t-test (n= 10,000 cells per experimental condition; ** *p*< 0.001). (I-J) Lysosomal membrane permeabilization analyzed by acridine orange (AO) (I) and Galectin-3 (J) assays, (J) *Down,* the number of LAMP1-positive Gal3 puncta was estimated from three independent experiments (n= 20 cells per experimental condition), with significant differences determined by Mann-Whitney (*****p*< 0.0001). Treatment with LLoMe (LMP inductor) for 30 min was used as positive control. (K) The analysis of lipofuscin aggregates was conducted in WT and HGPS-1/-2 cells grown on coverslips and treated with 60 nM Selinexor (Sel) or the vehicle alone for six days, using confocal microscopy at 550-650 nm. WT cells that were induced to senescence with 5 mM Sodium butyrate for ten days were used as positive control. Representative images are shown. The number of lipofuscin foci per cell was estimated from three independent experiments, with significant differences determined by Mann-Whitney (** *p*< 0.001; *****p*< 0.0001). (L) To evaluate the maturation of autophagosomes, the colocalization of LC3 and STX17 was analyzed by confocal microscopy. WT and HGPS-1 fibroblasts treated with 50 uM CQ or the vehicle for 24 h, prior to being cultured in KRB medium (starvation) for 60 min. The cells were then subjected to immunostaining using antibodies specific to STX17 or LC3, and counterstained with DAPI for the visualization of nuclei. *Right.* The Pearson correlation coefficient (PCC) for the STX17-LC3 colocalization was obtained from two independent experiments (n= 20 cells per experimental condition) using NIS elements software (Nikon), with significant differences determined by Mann-Whitney (** *p*= 0.0045). (M) To assess the fusion of autophagosomes and lysosomes, the colocalization of LC3 and LAMP1 was examined by confocal microscopy. WT and HGPS-1 cells were treated with 100 nM Bafilomycin A1 (BafA1) or the vehicle alone for 24 h prior to being subjected to double immunostaining for LC3 and LAMP1, and counterstaining with DAPI for nuclei visualization. *Right.* The number of LAMP1 positive LC3 puncta was quantified from two independent experiments (n= 20 cells per experiment), with significant differences being determined by Mann-Whitney (**** *p*= > 0.0001). (N**)** The effect of rescuing STX17 expression on autophagosome maturation was analyzed by confocal microscopy in CQ-treated HGPS fibroblasts grown under starvation. HGPS-1 cells were transduced with lentiviral vectors harboring an empty vector or a vector overexpressing STX17. After selection in neomycin-containing medium the fibroblasts were treated with 50 uM CQ or the vehicle for 24 h, prior to being cultured in KRB medium (starvation) for 60 min. The cells were then subjected to immunostaining using antibodies specific to STX17 and LC3, and counterstained with DAPI for the visualization of nuclei. *Right.* The Pearson correlation coefficient (PCC) for the STX17-LC3 colocalization was obtained from two independent experiments (n= 20 cells per experimental condition) using NIS elements software (Nikon), with significant differences determined by Mann-Whitney. (O) The impact of rescuing LAMP1 expression by lentiviral viral transduction on autophagosome-lysosome fusion was evaluated in HGPS-1 fibroblasts by confocal microscopy. HGPS-1 fibroblasts were transduced with lentiviral vectors harboring an empty vector or a vector overexpressing LAMP1. After selection in neomycin-containing medium the fibroblasts were treated with 100 nM Bafilomycin A1 (BafA1) or the vehicle alone for 24 h prior to being subjected to double immunostaining for LC3 and LAMP1, and counterstaining with DAPI for nuclei visualization. *Right.* The number of LAMP1 positive LC3 puncta was quantified from two independent experiments (n= 20 cells per experiment), with significant differences being determined by Mann-Whitney (**** *p*= > 0.0001). (P) The effect of Selinexor treatment on the LC3 and STX17 colocalization in HGPS fibroblasts. HGPS-1 fibroblasts were treated with 60 nM Selinexor for six days, and on the fifth day of treatment, the cells were incubated with CQ for 24 h and then transferred to KRB medium for 60 minutes (starvation), prior to undergoing immunostaining for colocalizing LC3 and STX17. Typical images from two biological replicates are shown. *Bottom.* The Pearson correlation coefficient (PCC) for the STX17-LC3 colocalization analysis was obtained from two independent experiments (n= 20 cells per experimental condition) using NIS elements software (Nikon), with significant differences determined by Mann-Whitney (** *p*= 0.0045).. (Q) The impact of Selinexor treatment on the colocalizing of LC3 and LAMP1 is shown. HGPS-1 fibroblasts were treated with 60 nM Selinexor or the vehicle alone for six days, and on the fifth day of treatment, the cells were incubated with 100 nM BafA1 (BAF) for 24 h and then transferred to KRB medium for 60 minutes (starvation), prior to undergoing immunostaining for colocalizing LC3 and LAMP1. The nuclei were stained with DAPI to enable their visualization. Representative images from two biological replicates are shown. *Right.* The quantity of LAMP1-positive LC3 puncta was determined from two separate experiments (n= 20 cells per experiment), with statistically significative differences being calculated by Mann-Whitney (**** *p*= < 0.0001). (R) Selinexor treatment induces the nuclear accumulation of TFEB. WT and HGPS-1 cells were treated with 60 nM Selinexor for six days, prior to be immunostaining with TFEB primary antibodies and counterstaining with DAPI to decorate the nuclei. Typical confocal images from three independent experiments are shown. *Right* The nuclear to cytoplasmic ratio of fluorescence (F n/c) was obtained, with significant differences being determined by Mann-Whitney (**** *p*< 0.0001). (S) Selinexor treatment induces the lysosomal activity in WT and HGPS cells. WT and HGPS-1 cells were treated with 60 nM Selinexor for six days, prior to analyze the lysosomal intracellular activity by staining the cells with a Lysosomal Intracellular Activity Kit and further analysis by flow cytometry. Statistically significant differences were determined from three independent experiments by unpaired t-test (n= 10,000 cells per experimental condition; * *p*= 0.0125 and ** *p*= 0.0011).

**Materials and methods**

**Cell culture and treatments**

The following human dermal fibroblast cultures were utilized. Skin fibroblasts from two different HGPS patients, an 8-year-old female donor (AG11513) and a 14-year-old female donor (AG01972), and from a healthy subject (AG08469), referred to as HGPS-1 and HGPS-2 and WT cell cultures respectively, were obtained from the Coriell Institute for Medical Research (Camden, NJ). BJ cells, a human dermal fibroblast cell line that contains a progerin construct, and human dermal fibroblasts (HDFs) containing doxycycline-inducible progerin or lamin A constructs [Kreienkamp R. et al., 2018]. All fibroblast cultures were grown in Minimal Essential Medium Eagle (MEM; Invitrogen, Carlsbad, CA), supplemented with 10% fetal bovine serum (FBS; Invitrogen, USA), 1 mM sodium pyruvate, antibiotics, and antimycotics (Sigma, Saint Louis, MO), at 37°C in a humidified 5% CO_2_ atmosphere incubator. In starvation conditions, fibroblast cultures were grown in Krebs-Ringer-Bicarbonate (KRB) buffer (Sigma, Saint Louis, MO) or grown for 12 h in MEM in the absence of FBS and sodium pyruvate. When indicated, fibroblast cultures were treated for 3 or 6 days with 60 nM selinexor (KPT-330, Kariopharm Therapeutics Inc.) diluted in DMSO to a <0.1% final concentration in the culture media. To halt autophagy, fibroblasts were treated for 12 h or 48 h with 50 µM Chloroquine (Sigma, Saint Louis, MO; C6628) or incubated with 10 µg/ml E64D and 10 µg/ml Pepstatin A for 4 h, prior to being subjected to WB analysis. All experiments were performed using fibroblast cultures at passage number 8-16.

**Antibodies**

The following primary antibodies were used: rabbit polyclonal and monoclonal antibodies against LC3 (MBL International, Woburn, MA [PM036]); LAMP1 (Sigma, Saint Louis, MO [L1418-200]); STX17 (Invitrogen, Carlsbad, CA, [PA5-40127]); TFEB (Cell Signaling Technology, MA [4240]); SQSTM1/p62 (Abcam, Cambridge, UK [Ab109012]); Galectin-3 (Abcam, Cambridge, UK [A3A12]); GFP (Santa Cruz Biotechnology, CA [sc-8334]). Mouse monoclonal antibodies against Progerin (Santa Cruz Biotechnology, CA [sc-81611]), and β-actin (a gift from Dr. Manuel Hernández, CINVESTAV, Mexico).

**Quantification of autophagic membranes by Cyto-ID dye**

To assess autophagic vacuoles (autophagosomes, and autolysosomes) in live cells, the fibroblasts were grown in p70 petri dishes (Sarstedt, Nümbrecht, Germany) until 80% confluency was reached. Thereafter, the cells were trypsinized and transferred to a 1.5-mL Eppendorf tube, to be stained with 1X of the CYTO-ID autophagy detection reagent (Enzo Life Sciences Farmingdale, NY) following the manufacturer´s instructions. Briefly, the cells were incubated for 1 hour at 37°C in the dark, then washed twice with PBS to remove the free dye and analyzed by flow cytometry at 488 nm (FL1 channel) using the BD Fortessa System. The data was plotted using Kaluza software. When indicated, the cells were treated with 50 µM CQ or PBS for 48 hours prior to analysis.

**Indirect Immunofluorescence and confocal microscope analysis**

Cells were seeded on coverslips and fixed with 4%paraformaldehyde (PFA) in phosphate-buffered saline (PBS) for 10 min. paraformaldehyde (PFA) in phosphate-buffered saline (PBS) for 10 min. Thereafter, they were permeabilized and blocked for 10 min at room temperature with immunofluorescence (IF) buffer containing 0.1% Triton X-100, 0.02% SDS, and 10 mg/ml BSA. Then, the cells were incubated at 4°C overnight with the corresponding primary antibodies. The following day, the cells were washed twice with PBS for 5 min, and then, incubated for 1 h at room temperature with the appropriate fluorochrome-conjugated secondary antibodies (Jackson Immuno Research Laboratories). Subsequently, the cells were incubated for 5 min at room temperature with DAPI (0.2 µg/ml diamino-2-phenylindole; Sigma-Aldrich) in PBS for nuclei labeling. After washing twice with PBS for 5 min, the coverslips were mounted on microscope slides using VectaShield (Vector Laboratories, Inc., Burlingame, CA) and further examined on a confocal laser scanning microscope (CLSM; Eclipse Ti-E inverted confocal laser scanning microscope, NiKon, Japan). The analysis of the digitized images was performed using ImageJ2 software, version: 2.14.0/1.54f (http://imageJ.nih.gov.ij).

**Lysobrite Red Lysosome Staining**

The fibroblasts were seeded in 35-mm dishes with glass bottoms in MEM medium at 70% confluency and cultured for 24 h. The cells were then incubated for 30 min at 37 °C in darkness with 1x LysoBrite^TM^ Red reagent (25157, Cayman Chemical, USA) and Hoechst (1 μg/mL) for lysosome and nuclei staining respectively. Following this, the medium was removed, and the cells were washed twice with PBS. Next, the cells were observed in an *in vivo* chamber at 37°C with 5% CO2. The images were captured using a laser confocal scanning microscope equipped with a ×63 objective lens (Eclipse Ti; Nikon, Japan) and processed using Fiji/ImageJ particle counter.

**Lysosomal membrane permeability (LMP) assays**

The fibroblasts were seeded onto 35-mm ss bottoms dishes in MEM medium with 10% of FBS and allowed to grow up to 80% confluency. The fibroblasts were then stained with 3 µg/ml orange acridine (AO) (Sigma, Saint Louis, MO [318337-1G]) for 15 min at 37°C in the dark. After the incubation period, the staining medium was aspirated, and the cells were washed twice with PBS 1X. Fresh culture medium was added, and the cells were imaged by confocal microscopy (Nikon Eclipse Ti), equipped with a CO₂ (5%) and temperature-controlled (37°C) device. Acridine orange was excited using laser lines to detect green fluorescence (cytoplasm/nuclei, 488 nm) and red fluorescence (acidic lysosomes, 543 nm) simultaneously. When indicated, a treatment for different times (0, 5, 30, and 60 min) with 1 mM L-Leucyl-L-Leucine methyl ester hydrobromide) (LLoMe; Sigma, Saint Louis, MO [L7393]) was used as inductor of LMP. The time-lapse images were acquired using a laser confocal scanning microscope equipped with a ×63 objective lens (Eclipse Ti; Nikon, Japan) Puncta quantification was performed using the Particle counter in Fiji/ImageJ to measure acidic/functional lysosomes. To analyze LMP by galaectin-3 assays, fibroblasts were immunostaining with antibodies against Galectin-3 and Lamp1, and further analyzed by indirect immunofluorescence and confocal microscopy, as described above.

**Lysosome Intracellular Activity**

The lysosomal activity of fibroblasts was measured using a lysosomal intracellular activity kit (ab234622, Abcam, Cambridge, UK), according to the manufacturer’s instructions. Briefly, fibroblasts were grown in p70 mm Petri dishes for 12 h in MEM medium supplemented with 10% FBS at 37°C with 5% CO_2_ The cells were then incubated in the dark with medium containing 1x self-quenched substrate for 1 h at 37°C with 5% CO_2_. When indicated, cells were pretreated with 1x Bafilomycin A1 (BA1; negative control) for 1 h, or with 60nM Selinexor or DMSO (vehicle) for six days prior to analysis. Cells were harvested and washed twice in 1 ml ice-cold 1× assay buffer and then resuspended in 300 µl PBS for analysis by flow cytometry (488-nm excitation laser) using the BD Fortessa System. The data was plotted using Kaluza software.

**Lipofuscin Assay**

The fibroblasts were grown on glass coverslips and cultured for 48 hours in MEM with 10% SFB medium at 37°C with 5% CO_2_ (70 – 80 % confluency). Autofluorescence of lipofuscin (aggregates of oxidized proteins and lipids) exhibits broad-spectrum autofluorescence, with peak emission wavelengths of 550 and 650 nanometers (nm). The cells were fixed with 4% paraformaldehyde (PFA) for 10 min and mounted on glass slides using Vecta shield (Vector Laboratories, Inc., Burlingame, CA) prior to being observed using an Axio Imager M2 microscope with Axiocam 506 mono camera (Carl Zeiss Microscopy GmbH, Jena, Germany) or Leica SP8 confocal microscope. A DsRed filter set was applied, with an exposure time of 500 milliseconds maintained throughout the observation. When indicated, cells were pretreated treated with Sodium Butyrate (5 mM) for 10 days (positive control) or with 60nM Selinexor or DMSO (vehicle) for six days, prior to analysis The foci of lipofuscin accumulation were subsequently measured using Fiji/ImageJ.

**Viral Transduction**

The retroviral transduction procedure was conducted as previously (Gonzalez-Suarez and Gonzalo, 2010). Briefly, HEK293T packing cells were transfected with a three-plasmid system (autophagic flux probe GFP-LC3-RFP-LC3ΔG, pUMVC3 and pVSV-G) using DNA X-tremeGENE HP (Roche). The autophagic flux probe GFP-LC3-RFP-LC3ΔG was purchased from Addgene (pMRX-IP-GFP-LC3-RFP-LC3ΔG, 84572). BJ cells expressing either an empty vector (EV) or Progerin were subjected to three rounds of transduction with retrovirus carrying the autophagic probe. Thereafter, the cells were selected in puromycin-containing medium (2 µg/ml). To rescue the expression of STAX17 and LAMP1, lentiviral transduction was carried out in WT and HGPS-1 fibroblasts as described above. The selection of transduced cells were performed in neomycin-containing medium. The following lentiviral vectors were used: pLV-Neo-CMV-ORF (empty vector), pLV-Neo-CMV-hSTX17 or pLV-Neo-CMV-hLAMP1 (Vector Builder; Chicago, IL. USA).

**Antibodies**

**Western Blotting**

The cells were resuspended in RIPA 1X buffer containing 1x Protease/Phosphatase inhibitor (Cell Signaling Technology, MA [5872S]) and 1% SDS. The lysates were obtained by sonication for 5 min and subsequently centrifugation at 14,000 rpm at 4°C for 10 min. Lysate samples were subjected to vertical electrophoresis on 12% SDS-polyacrylamide gels and transferred onto nitrocellulose membranes (Bio-Rad Laboratories, Hercules CA) using a semi-dry transblot apparatus (Bio-Rad Laboratories, Hercules CA) for 1 h or 15 min (for LC3 detection) at 20 volts. The membranes were blocked in TBST (100 mM Tris-HCl pH 8.0, 150 mM NaCl, 0.5% (v/v) Tween-20) with 5% low-fat dried milk for 1h at room temperature and further incubated at 4ºC overnight with the appropriate primary antibodies. Subsequently, the protein signal was developed using horseradish peroxidase-conjugate secondary antibodies (Sigma-Aldrich, MO, USA) and the enhanced chemiluminescence Western blotting detection system (Western Lightning, Plus ECL, Netherlands [NEL 104001EA]) according to the manufacturer´s instructions. The acquisition of the blot images was performed using the C-Digit^®^ Blot Scanner (LI-COR Biosciences, Lincoln, Nebraska). The digitized images were then subjected to analysis using Image Studio software, version: 5.2.5(LI-COR Biosciences, Lincoln, Nebraska).

**RNA Sequencing and Differential Expression Analysis**

Total RNA was extracted from HGPS-1 and WT cell lines, and vehicle treated and CQ-treated HGPS-1 and WT fibroblast cultures using standard phenol–chloroform standard method, followed by DNase I treatment to eliminate genomic DNA contamination. RNA integrity was assessed with the Agilent 2100 Bioanalyzer (Agilent Technologies), samples with RNA integrity number (RIN) ≥ 9.0 were used for sequencing. Library preparation for mRNA-seq was based on the Illumina TruSeq Stranded mRNA Library Prep Kit, following the manufacturer’s protocol. Library concentration and fragment size were evaluated using Qubit (Thermo Fisher) and Bioanalyzer. Sequencing was performed on the Illumina NovaSeq 6000 platform using paired-end mode (2 × 100 bp), generating an average of 30 million reads per sample without duplicates. Raw sequencing reads were assessed for quality using FastQC [Andrews 2010] (v0.11.9) with an average Phred score of 36, trimmed for adapters and low-quality bases with Trimmomatic [Anthony et al.,2014] (v0.39). Cleaned reads were aligned to the Homo sapiens reference genome (GRCh38) using STAR aligner [Dobin et al.,2013] (v2.7.10a). Read quantification at the gene level was performed with feature Counts [Liao et al.,2014] from the Subread package (v2.0.3). Downstream analysis was conducted in R (v4.2.3) using the edgeR package (v4.6.3) [Robinson et al., 2010]. Genes with low expression meaning counts per million <10 in at least two samples) were filtered out. Library normalization was carried out using the TMM (Trimmed Mean of M-values) method [Robinson & Oshlack, 2010]. Differential expression analysis was performed using a negative binomial model, and p-values were adjusted using the Benjamini–Hochberg method [Benjamini-Hochberg, 1995]. Genes with an adjusted p-value (FDR) < 0.0001 and |log₂ fold change| ≥ 1 were considered differentially expressed.

**Pathway and Functional Enrichment Analysis**

Gene set enrichment and pathway analysis were conducted using WEB-based GEne SeT AnaLysis Toolkit (WEBGESTALT) and PATHVIEW software (https://www.webgestalt.org/, https://bioconductor.org/packages/release/bioc/html/pathview.html ). Differentially expressed genes (DEGs) identified from the RNA-seq analysis were uploaded into WEBGESTALT for core analysis, using the molecular processes database as the reference dataset. The analysis included prediction of reported and enriched canonical pathways, upstream regulators, biological functions, and disease associations, based on curated and validated interactions and experimental data reports. Significance of pathway enrichment was determined using two methods for identifying enriched categories that will be identified based on FDR threshold and TOP means the categories will be first ranked based on the FDR and then the TOPMOST significant categories will be selected. For ORA analysis WebGestalt will reflect the topmost significant categories from each of positive and negative related categories and was determined using the Fisher’s exact test. Only genes with an adjusted p-value (FDR) < 0.5 and absolute log₂ fold change ≥ 1 were used in the analysis. For the autophagy and lysosomal gene lists, genes with an FDR-adjusted P value ≤ 0.05 were considered differentially expressed genes (DEGs)

**Statistical analysis**

Statistical analyses were performed using GraphPad Prism 9 software (San Diego) by the two‐tailed unpaired Student's t-test. Data represent the mean ± SEM from three independent experiments, and *p* values ˂ 0.05 are indicative of statistical significance. Where indicated, statistical analyses were performed using exact nonparametric Mann-Whitney U test or unpaired t-test, and data were represented by the mean ± SEM or the mean ± SD from a series of three independent experiments, *p* values ˂ 0.05 were considered as significant.
